# Stage‐Associated Collagen Gene Expression and Macrophage‐Associated Immune Features in Nail Unit Melanoma

**DOI:** 10.1111/pcmr.70143

**Published:** 2026-11-11

**Authors:** Yuri Sawada, Marco A. De Velasco, Megumi Aoki, Kazuko Sakai, Naoki Shiraishi, Kazuyasu Fujii, Shigeto Matsushita, Kazuto Nishio, Atsushi Otsuka

**Affiliations:** ^1^ Department of Dermatology Kindai University Faculty of Medicine Sakai Osaka Japan; ^2^ Department of Genome Biology Kindai University Faculty of Medicine Sakai Osaka Japan; ^3^ Department of Dermato‐Oncology NHO Kagoshima Medical Center Kagoshima City Kagoshima Japan; ^4^ Center for Genomics, Life Science Research Institute Kindai University Sakai Osaka Japan; ^5^ Genome Medical Center, Kindai University Hospital Sakai Osaka Japan

**Keywords:** collagen, extracellular matrix, gene expression profiling, melanoma, nail diseases, tumor microenvironment, tumor‐associated macrophages

## Abstract

Acral melanoma, a rare subtype arising on the palms, soles, and nail unit, responds poorly to immune checkpoint inhibitors. Among acral melanomas, nail unit melanoma is less common than plantar melanoma, and the transcriptomic programs associated with its progression remain poorly understood. We performed bulk RNA sequencing of 43 nail unit melanoma specimens from early‐ to late‐stage disease. Unsupervised clustering identified two transcriptomic groups that were associated with clinical stage, with one group enriched for late‐stage tumors and the other for early‐stage tumors. The late‐stage‐enriched group showed higher expression of collagen/extracellular matrix (ECM) genes involved in collagen processing, assembly, and remodeling, whereas the early‐stage‐enriched group showed higher expression of epidermal and basement membrane‐associated genes. In the late‐stage‐enriched group, deconvolution showed higher macrophage and lower CD8+ T cell estimates, and *SPP1* and *APOE* were more highly expressed; both genes also showed higher expression in late‐stage tumors. Across tumors, macrophage and tumor‐associated macrophage signature scores correlated positively with a collagen biosynthesis gene‐set score, including after adjustment for histological tumor purity. Several stage‐associated differences were attenuated after purity adjustment. These tissue‐level associations describe collagen/ECM‐ and macrophage‐associated features of nail unit melanoma; their cellular origin and clinical relevance require independent evaluation.

## Abstract (highlights)

**Significance:**

Acral melanoma, including nail unit melanoma, responds poorly to immune checkpoint inhibitors, and the tissue features that accompany the clinical stage of nail unit melanoma are not well characterized. In this bulk transcriptomic study of an understudied melanoma subtype, the late‐stage‐enriched group showed higher expression of collagen/extracellular matrix genes and higher macrophage estimates. Across tumors, macrophage‐associated signature scores correlated with a collagen biosynthesis gene‐set score. These findings provide a descriptive basis for studying the collagen/extracellular matrix and myeloid compartments of nail unit melanoma with spatial, single‐cell, and clinical approaches.

**Research Highlights:**

Nail melanomas show stage‐associated collagen gene expression. A late‐stage‐enriched group has higher macrophage and lower CD8 T‐cell estimates. Macrophage signature scores correlate with a collagen gene‐set score.

## Graphical abstract

Bulk RNA sequencing of 43 formalin‐fixed, paraffin‐embedded nail unit melanomas identified two transcriptomic groups associated with clinical stage. The early‐stage‐enriched group showed higher expression of epidermal adhesion and keratinocyte differentiation genes, whereas the late‐stage‐enriched group showed higher expression of melanocytic and collagen/extracellular matrix‐stromal genes, inflammatory myeloid genes, and chromatin and interferon‐γ/MHC antigen presentation genes.

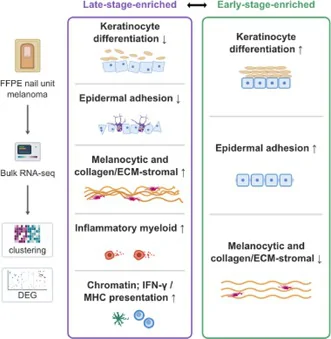

## 1 Introduction

Nail unit melanoma is a rare form of acral melanoma that arises from the melanocytes within the nail apparatus (Tan et al. 2007). In US registry data, acral lentiginous melanoma accounted for a larger proportion of melanomas in Black and Asian/Pacific Islander populations than in non‐Hispanic White populations (Bradford et al. 2009). Diagnosis is frequently delayed because early lesions resemble benign melanonychia, so that many patients present with advanced disease and show a poor prognosis (Lieberherr et al. 2020).

Acral melanoma, including the nail unit subtype, is etiologically and genetically distinct from cutaneous melanoma that is associated with sun exposure. It harbors a markedly lower tumor mutational burden, infrequently carries *BRAF* mutations, and shows a relatively high frequency of *KIT* alterations and structural genomic changes (Newell et al. 2020). Although ultraviolet (UV) radiation is not considered a major driver of acral melanoma overall, UV‐associated mutational signatures have been identified in a subset of subungual tumors (Newell et al. 2020). Clinically, acral melanoma responds poorly to immune checkpoint inhibitor therapy (Nakamura et al. 2020; van Not et al. 2022); in the primary study cohort (Nakamura et al. 2020), patients with nail unit melanoma had poorer treatment responses and survival outcomes than those with palm or sole melanoma. This limited efficacy has previously been reported as being associated with a non–T cell‐inflamed tumor microenvironment (TME) with low CD8+ T cell infiltration and low PD‐L1 expression (Augustin et al. 2023). In a cohort of patients with metastatic melanoma, the association between tumor mutational burden and response to anti‐PD1 therapy was confounded by melanoma subtype (Liu et al. 2019). The TME programs that accompany progression of nail unit melanomas, and how they might contribute to immune checkpoint inhibitor resistance, have not been systematically defined.

Remodeling of the extracellular matrix (ECM), in particular, desmoplastic deposition and cross‐linking of collagen, has been demonstrated to be associated with an immunosuppressive microenvironment in several malignancies (Tharp et al. 2024). Whether collagen/ECM‐related gene expression is associated with immune features in nail unit melanoma, however, has not yet been examined.

We profiled 43 nail unit melanomas spanning early‐ to late‐stage disease using bulk RNA sequencing, unsupervised clustering, co‐expression module analysis, and immune cell deconvolution to examine stage‐associated collagen/ECM expression, immune, and stromal features, and their associations.

## 2 Materials and Methods

### 2.1 Patient Cohort and Tissue Collection

A total of 43 patients with nail unit melanoma from two institutions, namely, the NHO Kagoshima Medical Center and Kindai University Hospital, were enrolled in this study. Formalin‐fixed, paraffin‐embedded (FFPE) specimens prepared from surgically resected primary tumors were collected (Table 1). The malignancy was classified as early stage (Stage 0–IIA; *n* = 22) or late stage (Stage IIB or higher; *n* = 21) in accordance with the American Joint Committee on Cancer (AJCC), 8th edition. For the cross‐subtype comparison, an additional cohort with late‐stage disease (Stage IIB or higher) was enrolled, including six patients with plantar acral melanoma and six cases with nonacral cutaneous melanoma. The study was reviewed and approved by the Ethics Committee of the Faculty of Medicine, Kindai University, with the implementation authorization of the Dean of the Faculty of Medicine; the use and provision of archival specimens at the participating institutions were conducted in accordance with the applicable institutional ethical procedures. All procedures were performed in accordance with the Declaration of Helsinki. Written informed consent from the patients was obtained when required; for archival specimens, the need for consent was waived under an approved opt‐out procedure. Histological tumor purity was assessed on hematoxylin and eosin‐stained sections: a pathologist marked the tumor area, which was macrodissected for RNA extraction, a dermatologist reviewed and agreed with the assessment, and purity was recorded as the proportion of tumor cells within the marked area.

**TABLE 1:** Clinicopathological characteristics of the nail unit melanoma cohort (*n* = 43).

Characteristic	Total (*n* = 43)	Early stage (*n* = 22)	Late stage (*n* = 21)
Age, years, median (range)	75 (12–90)	75 (12–90)	78 (39–90)
**Sex, *n* (%)**			
Female	23 (53)	13 (59)	10 (48)
Male	20 (47)	9 (41)	11 (52)
**Anatomic subsite, *n* (%)**			
Fingernail	29 (67)	17 (77)	12 (57)
Toenail	14 (33)	5 (23)	9 (43)
Breslow thickness, mm, median (range)^a^	2.5 (0.3–15.0)	0.6 (0.4–1.8)	3.9 (0.3–15.0)
**AJCC T category, *n* (%)**			
Tis	15 (35)	15 (68)	0 (0)
T1	6 (14)	5 (23)	1 (5)
T2	2 (5)	2 (9)	0 (0)
T3	9 (21)	0 (0)	9 (43)
T4	11 (26)	0 (0)	11 (52)
**AJCC N category, *n* (%)**			
N0	35 (81)	22 (100)	13 (62)
N1	4 (9)	0 (0)	4 (19)
N2	2 (5)	0 (0)	2 (10)
N3	2 (5)	0 (0)	2 (10)
**AJCC M category, *n* (%)**			
M0	42 (98)	22 (100)	20 (95)
M1	1 (2)	0 (0)	1 (5)
**AJCC stage group, *n* (%)**			
0	15 (35)	15 (68)	0 (0)
I	5 (12)	5 (23)	0 (0)
II	15 (35)	2 (9)	13 (62)
III	7 (16)	0 (0)	7 (33)
IV	1 (2)	0 (0)	1 (5)
Tumor purity, %, median (range)	30 (10–80)	20 (10–40)	60 (10–80)
**Hierarchical cluster, *n* (%)** ^b^			
HC1	17 (40)	2 (9)	15 (71)
HC2	26 (60)	20 (91)	6 (29)
**Louvain cluster, *n* (%)** ^b^			
C1	17 (40)	2 (9)	15 (71)
C2	13 (30)	9 (41)	4 (19)
C3	12 (28)	10 (45)	2 (10)
C4	1 (2)	1 (5)	0 (0)

*Note:* Early stage, AJCC stage 0–IIA; late stage, AJCC Stage IIB or higher (AJCC 8th edition). Percentages are column percentages and may not add up to 100 owing to rounding.

Abbreviations: AJCC, American Joint Committee on Cancer; HC, hierarchical cluster.

^a^ Breslow thickness summarized for invasive tumors with available measurement (*n* = 27); in situ (Tis) tumors (*n* = 15) were excluded, and the thickness was not available for one invasive tumor.

^b^ Hierarchical clusters (HC1, HC2) and Louvain clusters (C1–C4) were derived from unsupervised transcriptomic analysis and are shown for reference; they are not clinicopathological variables.

### 2.2 RNA Extraction and Bulk RNA Sequencing

Total RNA was extracted from the FFPE sections using the AllPrep DNA/RNA FFPE Kit (QIAGEN, Hilden, Germany). Library preparation and sequencing were performed by Takara Bio Inc. Gene Analysis Center (Kusatsu, Japan). Paired‐end reads were aligned to the human reference genome (GRCh38) using STAR (RRID:SCR_004463), and gene‐level read counts were obtained using featureCounts (RRID:SCR_012919; Subread package, RRID:SCR_009803) with the GENCODE v39 primary assembly annotation. Counts were normalized by the trimmed mean of M‐values (TMM) method (edgeR, RRID:SCR_012802; R 4.5.0, RRID:SCR_001905). Unless otherwise stated, downstream analyses were performed using protein‐coding genes (GENCODE v39 biotype) with expression quantified as log_2_ counts per million (CPM), computed with edgeR (log_2_, prior.count = 1); immune cell deconvolution included all genes, regardless of the biotype.

### 2.3 Statistical Analysis

All statistical analyses were performed using R version 4.5.0 (R Project for Statistical Computing). Unless otherwise stated, all tests were two‐sided, and *p* values from multiple comparisons were adjusted by the Benjamini–Hochberg (BH) method; a BH‐adjusted *p* (*p*_adj_) < 0.05 was considered statistically significant. In the text and figures, *p* values are reported to three decimal places; values < 0.001 are shown as “< 0.001”. Supplementary tables retain the numerical precision of the saved outputs. Wilcoxon rank‐sum tests used the normal approximation with continuity correction unless described as exact, and their effect sizes are reported as rank‐biserial *r*. In box plots, boxes show the median and interquartile range, whiskers extend to the most extreme values within 1.5 times the interquartile range from the box, and each dot is one tumor. Significance thresholds: ***p_adj_ < 0.001; **p_adj_ < 0.01; *p_adj_ < 0.05; ns, not significant.

### 2.4 Differential Expression Analysis

Genes were filtered using filterByExpr (edgeR; min.count = 10, min.total.count = 15, large.n = 10, min.prop = 0.7) to define the gene universe. Differentially expressed genes between late‐stage and early‐stage tumors, and between HC1 and HC2 tumors were assessed with the limma–voom pipeline (limma, RRID:SCR_010943) using voomWithQualityWeights, after adjustments for sequencing batch and patient age (primary model). Because tumor purity (a histological estimate; Table 1) was correlated with the early/late stage indicator (Pearson *r* = 0.633), it was excluded from the primary model to avoid removing genuine stage‐associated signals, and a sensitivity model additionally included histological tumor purity. An additional model included ESTIMATE‐derived purity in place of histological purity; this expression‐derived measure reflects stromal and immune signals and was not treated as an independent replication of histological purity. A reduced model included only the group and histological tumor purity, without age or sequencing batch, and is reported for completeness. A focused set of 91 collagen‐related genes was examined. It comprised (i) all 44 collagen alpha‐chain genes in the analyzed gene universe, identified by the gene‐symbol rule ^COL[0‐9]+A[0‐9]+$; (ii) a fixed list of 26 genes involved in collagen biosynthesis, post‐translational modification, cross‐linking, chaperoning and processing, grouped into six functional classes (lysyl hydroxylases, prolyl 4‐hydroxylases, lysyl oxidases, prolyl 3‐hydroxylation complex, chaperones and procollagen‐processing enzymes, and collagen glycosyltransferases); and (iii) 21 genes annotated to collagen‐related terms (all terms whose names contain “collagen”) in the Metascape enrichment analysis of the co‐expression modules. Differential expression of these genes was taken from the genome‐wide analysis (correction across all tested genes), with and without adjustment for histological tumor purity. For the collagen/ECM genes that met the HC1‐versus‐HC2 criteria (*p*_adj_ < 0.05 and |log_2_ fold change| ≥ 1), voom log_2_ CPM values were also compared by the Wilcoxon rank‐sum test with correction across these genes, separately for each axis.

### 2.5 Unsupervised Transcriptomic Clustering

Genes were ranked by variance of log_2_(transcripts per million (TPM) + 1) across all 43 samples, and the top 2000 genes with the highest variances were selected for unsupervised analysis. Louvain clustering was first applied to these genes using Orange3 (version 3.38.1, RRID:SCR_019811), with principal component analysis (PCA) preprocessing (7 principal components, without normalization), a *k*‐nearest‐neighbor graph with *k* = 14 neighbors, and Euclidean distance; this yielded four clusters (C1–C4), one of which (C4) contained a single sample. The samples were visualized by t‐distributed stochastic neighbor embedding (t‐SNE) in Orange3 (PCA preprocessing with 5 principal components, PCA initialization, Euclidean distance, perplexity 30, exaggeration 1). Unsupervised hierarchical clustering of the samples was then performed in Morpheus (Broad Institute, RRID:SCR_017386) on gene‐wise z‐scored values of these genes using one minus Pearson correlation as the distance metric and complete linkage, which yielded two major clusters that we designated as HC1 and HC2. The hierarchical partition was used for all downstream analyses because its two groups correspond to the first split of the dendrogram and are both large enough for group comparisons, whereas Louvain clustering yielded a cluster containing a single sample; the Louvain assignments were displayed as an annotation in the hierarchical clustering heatmap, and their correspondence with HC1/HC2 was summarized by the adjusted Rand index. Cluster quality was assessed by the average silhouette width for *k* = 2–5, and cluster stability by bootstrap resampling (*B* = 1000; clusterwise Jaccard coefficient), both with the fixed set of 2000 genes and with re‐selection of the 2000 most variable genes within each iteration. The association between cluster membership (HC1/HC2) and clinical stage (early/late) was assessed by the *χ* ^2^ test with Yates' continuity correction and Fisher's exact test; the odds ratio was derived from the 2 × 2 contingency table.

### 2.6 Co‐Expression Module Definition, Annotation, Scoring, and Inter‐Module Network Analysis

Co‐expression modules (M1–M10) were defined by cutting the gene dendrogram obtained from hierarchical clustering (one minus Pearson correlation of gene‐wise *z*‐scored values, complete linkage) at a level that yielded 10 groups (*k* = 10). This cut level was chosen on an exploratory basis, informed by the biological interpretability of the resulting groups. Each module was functionally annotated in Metascape (Express Analysis; RRID:SCR_016620) (Zhou et al. 2019) by over‐representation analysis (ORA) and protein–protein interaction (PPI) network analysis; PPI networks were built from STRING (physical score > 0.132), BioGrid (physical), OmniPath, and InWeb_IM, and densely connected components were detected by MCODE (RRID:SCR_015828) with the Metascape default settings. Module and component labels were assigned by the authors with reference to the enrichment results and member genes. For each sample, a module score was computed as the mean *z*‐score of the member genes of the module. Module scores were compared between HC1 and HC2, and between late‐ and early‐stage tumors, by the exact Wilcoxon rank‐sum test with correction across the 10 modules within each comparison. Inter‐module relationships were assessed by Spearman correlations (*ρ*) between the 10 module scores (45 pairs) across all 43 samples. A co‐expression network was constructed with modules as nodes and correlations with BH‐adjusted *p* < 0.05 (adjusted across the 45 pairs) as edges. Network centrality was quantified as the ρ‐weighted eigenvector centrality on the subgraph of significant positive edges, rescaled to a maximum of 1 (igraph v2.1.4, RRID:SCR_021238). To assess dependence on the dominant axis of variation, partial Spearman correlations between module scores were computed using the ppcor package, conditioning on HC group, stage, both, or the first principal component of the gene‐wise *z*‐scored expression matrix of the 2000 most variable genes, with correction across the 45 pairs within each condition; an edge was considered retained when |*ρ*| ≥ 0.3 with the same sign before and after conditioning.

The robustness of the module definitions was examined in four ways: (i) the gene dendrogram was cut at *k* = 6–14 instead of 10; (ii) the 500–5000 most variable genes were clustered and cut at *k* = 10 and the resulting clusters were compared with the original modules; (iii) samples were resampled 200 times, the gene dendrogram was recomputed from the distinct samples drawn in each resample, and each original module was matched to the resampled cluster with the highest Jaccard index (stability of module boundaries); and (iv) samples were resampled 500 times with the module definitions, scores, and HC labels held fixed, and the proportion of resamples reproducing the sign of the HC1–HC2 difference was recorded. In addition, the genes of each module were randomly split into halves 1000 times, and the correlation between half‐module scores was computed as a descriptive reference for the inter‐module correlations.

### 2.7 Gene Set Enrichment Analysis

GSEA was performed using GSEA version 4.4.0 (Broad Institute, RRID:SCR_003199) with the HALLMARK gene set collection from MSigDB. Significance was assessed by sample permutation, and gene sets with false discovery rate (FDR) *q* < 0.25, the conventional threshold for GSEA, were considered enriched. For comparisons across the four limma models described above (primary, histological purity‐adjusted, ESTIMATE purity‐adjusted, and reduced), preranked enrichment analysis used limma moderated t statistics for 17,483 mapped genes and the 50 HALLMARK gene sets (MSigDB v2026.1.Hs), with fgseaMultilevel (minSize = 15, maxSize = 500, eps = 1e‐10). *p* values were adjusted across the 50 sets separately for each comparison and model, using *p*_adj_ < 0.05. This analysis was distinct from the sample‐permutation GSEA described above.

### 2.8 Immune Cell Deconvolution

Tumor immune cell infiltration was estimated using TIMER3 (RRID:SCR_018737) (Cui et al. 2025), which aggregates scores from multiple deconvolution algorithms (TIMER, CIBERSORT/CIBERSORT‐ABS, quanTIseq, xCell, MCP‐counter, EPIC, ConsensusTME, ABIS, ESTIMATE, and ImmuCellAI) (Aran et al. 2017; Becht et al. 2016; Finotello et al. 2019; Jiménez‐Sánchez et al. 2019; Li et al. 2016; Miao et al. 2020; Monaco et al. 2019; Newman et al. 2015; Racle et al. 2017; Yoshihara et al. 2013); scores were used directly from the TIMER3 output. For each cell type, differences between HC1 and HC2 for each algorithm were tested using the Wilcoxon rank‐sum test, with correction across all 182 estimates. Cell types showing significant (BH‐adjusted *p* < 0.05), directionally concordant differences in at least two algorithms were defined as consistently altered and are reported below. CIBERSORT relative estimates describe composition within the reference cell populations rather than absolute abundance in the tissue. Only CIBERSORT‐ABS was counted toward the concordance criterion, to avoid counting two modes of the same method; both modes were retained in the correction across all 182 estimates. Cell‐type labels that differed only in spelling between algorithms were harmonized. The same analyses were performed for late‐ versus early‐stage tumors.

### 2.9 Gene Signature Scores

Gene signature scores were calculated from TMM‐normalized log_2_ CPM without expression filtering: expression values of each gene were *z*‐scored across all 43 samples and averaged per sample. Genes with zero counts in all samples or zero variance were excluded, and genes absent from the expression data were omitted. Externally defined signatures were used for macrophages and CD8 T cells (Danaher et al. 2017) and for B cells and tumor‐associated macrophages (Bagaev et al. 2021). These signatures were compared between HC1 and HC2 and between late‐ and early‐stage tumors by the Wilcoxon rank‐sum test with correction across the four signatures within each comparison; 24 macrophage‐associated genes selected by the authors (Table S6D), including *SPP1* and *APOE*, were compared in the same way with correction across the 24 genes. Associations of the macrophage and tumor‐associated macrophage signature scores and of *SPP1* and *APOE* expression with the Reactome collagen biosynthesis and modifying enzymes gene‐set score (67 genes) were assessed across all 43 samples by Spearman correlation, with percentile bootstrap 95% confidence intervals (2000 resamples), and by partial Spearman correlation adjusted for histological tumor purity (all variables rank‐transformed; *t*‐test), with 95% confidence intervals from the Fisher *z* approximation; correction was applied across the four tests within each analysis. T‐cell states were assessed with an activated CD8 T‐cell signature of 26 genes (Charoentong et al. 2017) and an inhibitory receptor signature (*PDCD1*, *CTLA4*, *LAG3*, *TIGIT*, and *HAVCR2*) selected in this study from the checkpoint genes of the immunophenoscore (Charoentong et al. 2017), by the Wilcoxon rank‐sum test and by linear models of the score on group adjusted for log_2_(MCP‐counter CD8+ T‐cell estimate +1) (*t*‐test of the group coefficient), with correction across the two signatures within each comparison and analysis.

Collagen gene‐set scores were calculated for three externally defined gene sets (MSigDB 2025.1.Hs; msigdbr 25.1.1): Reactome collagen biosynthesis and modifying enzymes, NABA collagens, and NABA core matrisome. Scores were also calculated for versions of these sets without the genes shared with a 14‐gene score, and for the 14‐gene score itself. The 14‐gene score comprised the 14 of the 18 HC1‐enriched collagen/ECM genes that are involved in collagen biosynthesis and maturation (*ADAMTS3*, *BMP1*, *COL9A1*, *COL9A3*, *COL11A1*, *COL11A2*, *COL19A1*, *COL22A1*, *COLGALT2*, *FKBP10*, *LOXL2*, *PLOD1*, *PLOD3*, and *SERPINH1*). Because its genes were selected with reference to the HC comparison, it was used only as a descriptive reference and not as independent evidence. Stage comparisons used Wilcoxon rank‐sum tests and linear models adjusted for age and sequencing batch, with additional models including histological tumor purity; T category (Tis = 0, T1–T4 = 1–4) was analyzed by Spearman correlation and linear models with the same covariates. Correction was applied across the seven scores for each axis and test or model; the descriptive HC comparison was restricted to four scores.

In sensitivity analyses for tumor purity, selected collagen‐ and macrophage‐related genes and the 14‐gene score were analyzed by ordinary linear models (*t*‐tests of the coefficient) of each marker on group, stage, or T category, without adjustment, with adjustment for age and sequencing batch, and additionally with histological tumor purity, with correction across six markers for each exposure and model. The association between histological and ESTIMATE‐derived tumor purity was assessed by Spearman correlation with percentile bootstrap 95% confidence intervals (2000 resamples). The association between the MCP‐counter fibroblast estimate and a Reactome collagen biosynthesis score from which the four MCP‐counter fibroblast marker genes were removed (63 genes) was assessed as a single prespecified Spearman correlation.

### 2.10 Cross‐Subtype Comparison

Late‐stage nail unit melanoma (*n* = 21) was compared with late‐stage nonnail melanoma (plantar acral, *n* = 6; nonacral cutaneous, *n* = 6) for the 35 collagen/ECM genes that met the HC1‐versus‐HC2 criteria, two collagen scores, and 12 immune cell estimates. The two collagen scores were the mean per‐gene *z*‐scores of the 18 HC1‐enriched and the 17 HC2‐enriched genes, calculated across these 33 tumors. Nail unit and nonnail melanoma were compared by the Wilcoxon rank‐sum test, and the three groups by the Kruskal–Wallis test, each with correction across the 37 genes and scores or the 12 cell‐type estimates. Pairwise Dunn tests with correction for ties were corrected across the three pairs and were interpreted only when the Kruskal–Wallis test was significant. Values were rounded to 12 significant digits before ranking so that values identical except for floating‐point error were treated as ties.

### 2.11 Nail Subsite

Differences between fingernail and toenail lesions were assessed by linear models of each score on subsite adjusted for lesion category (in situ [stage 0], Stage I–IIA, or Stage IIB or higher), age, and sequencing batch, with an additional model including histological tumor purity; correction was applied across 10 scores within each model. Analyses restricted to late‐stage lesions (score on subsite) were descriptive, with unadjusted *p* values. The distribution of subsites between early and late stage was assessed by Fisher's exact test.

## 3 Results

### 3.1 Unsupervised Transcriptomic Clustering Separated Nail Unit Melanomas Into Groups Associated With Clinical Stage

We profiled nail unit melanomas across clinical stages (Figure 1A). Genome‐wide examination of differentially expressed genes between early‐ and late‐stage tumors revealed upregulation in late‐stage tumors of melanocytic transcription factors (*SOX10*, *MITF*), the inflammatory chemokine *CXCL8*, tumor‐microenvironment‐associated genes (*SPP1*, *APOE*), and collagen/ECM‐remodeling genes (*COL11A1*), and enrichment of epidermal and stromal genes such as *COL17A1* in early‐stage tumors (Figure 1B; Table S1A). In a sensitivity model additionally adjusted for histological tumor purity, the number of genes with *p*_adj_ < 0.05 decreased from 7785 to 33 for the stage comparison and from 9573 to 855 for the HC1‐versus‐HC2 comparison. Among genes significant in the primary model, the primary and purity‐adjusted log_2_ fold changes remained correlated (Pearson *r* = 0.814 for stage and 0.919 for HC), but regression slopes of the adjusted on the primary estimates were 0.460 and 0.714, respectively, indicating attenuation of effect sizes. Within these gene sets, 494 and 547 genes, respectively, changed the sign of their log_2_ fold change (Table S1C–E). Because purity was correlated with stage, this attenuation does not by itself show that the stage differences are attributable to tissue composition.

**FIGURE 1:**
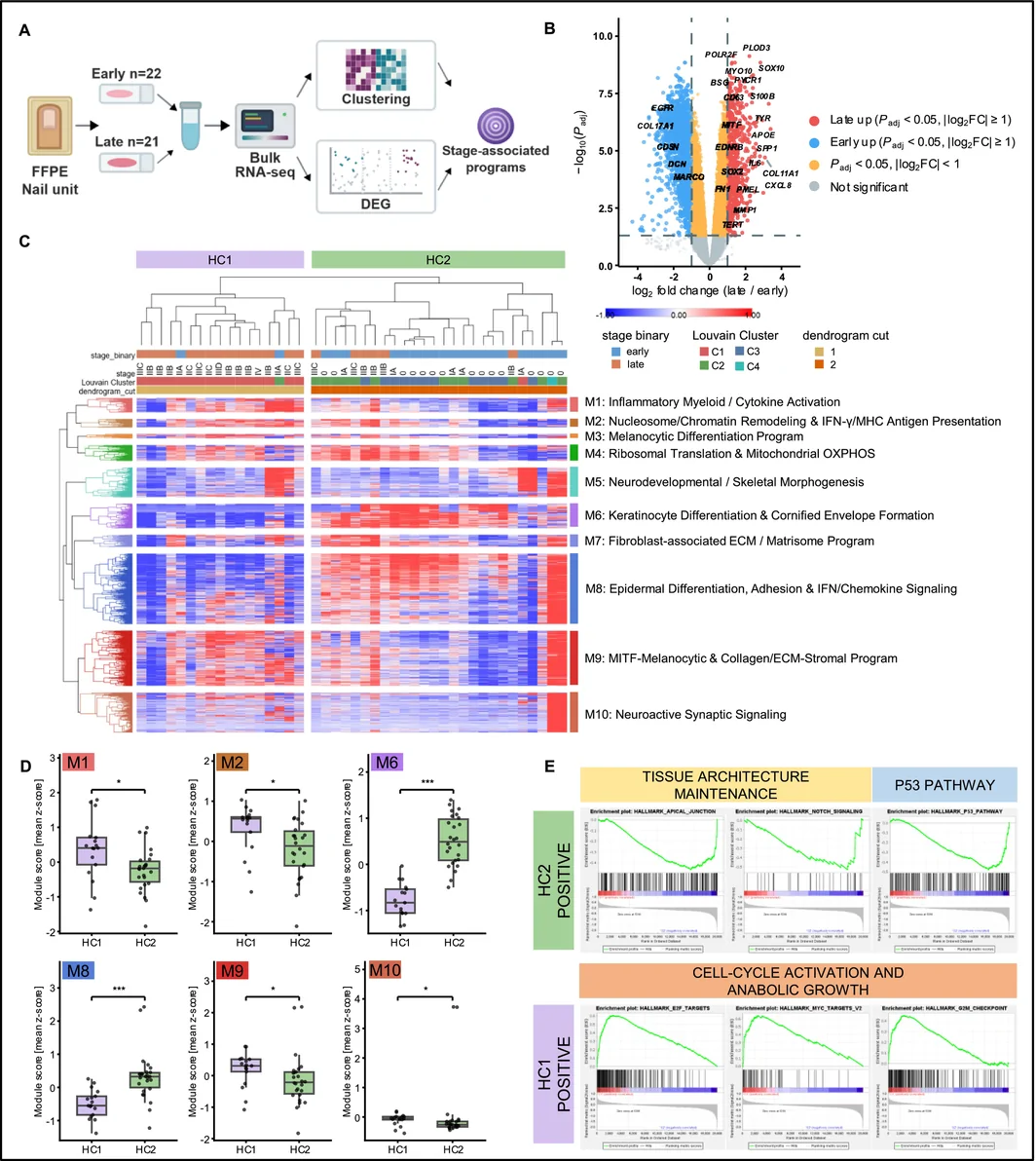
Unsupervised transcriptomic clustering of nail unit melanoma reveals stage‐associated gene expression modules. (A) Study workflow. Bulk RNA sequencing of formalin‐fixed, paraffin‐embedded (FFPE) nail unit melanoma specimens (early stage, *n* = 22; late stage, *n* = 21) was analyzed by unsupervised clustering and differential expression analysis to identify stage‐associated transcriptional programs. (B) Volcano plot of differential expression between late‐ and early‐stage tumors (limma–voom; model ~ stage + age + sequencing batch; Benjamini–Hochberg (BH) correction across 17,485 genes; Table S1A). Red, higher in late‐stage tumors; blue, higher in early‐stage tumors (*p*_adj_ < 0.05 and |log_2_ fold change| ≥ 1); orange, *p*_adj_ < 0.05 with |log_2_ fold change| < 1; gray, not significant. (C) Heatmap of gene‐wise *z*‐scores of the 2000 most variable genes. Samples (columns) were ordered by unsupervised hierarchical clustering (1 − Pearson correlation, complete linkage); the first split of the sample dendrogram defined two hierarchical clustering (HC) clusters, HC1 and HC2 (cluster quality and stability, Figure S1 and Table S2E,F). Column annotation bars: Stage_binary (early, AJCC stage 0–IIA; late, Stage IIB or higher); stage (AJCC stage); Louvain Cluster (Louvain clusters C1–C4; Figure S1A and Table S2D); and dendrogram_cut (HC1/HC2 assignment). Rows are grouped into co‐expression modules M1–M10 (Table S2C), with functional annotations on the right. (D) Module scores (mean gene‐wise *z*‐score) of M1, M2, M6, M8, M9, and M10 in HC1 versus HC2; the other four modules are shown in Figure S2A. Two‐sided Wilcoxon rank‐sum test (exact) with BH correction across the 10 modules; effect size, rank‐biserial *r* (Table S2A). (E) Selected HALLMARK gene‐set enrichment plots for HC1 versus HC2, obtained by gene set enrichment analysis (GSEA) with sample permutation. Enrichment was assessed using the GSEA false discovery rate (FDR *q* < 0.25) across the HALLMARK collection (50 gene sets; Table S3A). Functional grouping labels were assigned by the authors. In (D), asterisks denote *p*_adj_ (*, < 0.05; **, < 0.01; ***, < 0.001). HC1, *n* = 17; HC2, *n* = 26.

Louvain clustering of the 43 tumors based on the expression of the 2000 most variably expressed genes yielded four clusters (C1–C4), one of which (C4) contained a single tumor (Figure S1A). C1 comprised predominantly late‐stage tumors (15/17), whereas C2–C4 comprised predominantly early‐stage tumors (20/26) (Table 1). Unsupervised hierarchical clustering of the same tumors using the same expression data, with the Louvain assignments displayed as an annotation (Figure 1C), partitioned the 43 tumors into two clusters, HC1 (*n* = 17) and HC2 (*n* = 26), which were strongly associated with the clinical stage: HC1 comprised predominantly late‐stage tumors (15/17, 88%; early‐stage tumors, 2/17, 12%), whereas HC2 comprised predominantly early‐stage tumors (20/26, 77%; late‐stage tumors, 6/26, 23%) (*χ* ^2^ = 14.955 with Yates' correction, *p* < 0.001; Fisher's exact *p* < 0.001; odds ratio = 25.0). The correspondence between the two partitions was quantified: C1 contained 16 of the 17 HC1 tumors, and 25 of the 26 HC2 tumors were assigned to C2–C4. The adjusted Rand index was 0.479 for HC1/HC2 versus C1–C4 and 0.818 after combining C2–C4 into one group (Table S2D). Because both partitions were derived from the same expression data, this correspondence is a consistency check rather than independent validation. The two‐group hierarchical solution had the highest average silhouette width among *k* = 2–5 (0.318, with four of 43 samples showing negative widths; Figure S1B,C; Table S2E), indicating a modest separation, and the partition was stable under bootstrap resampling (mean clusterwise Jaccard coefficient 0.760 for HC1 and 0.804 for HC2 with the 2000 genes fixed, and 0.739 and 0.791 with the variable genes re‐selected in each resample; Figure S1D; Table S2F). The hierarchical partition was used for downstream analyses (Methods). Hereafter, HC1 and HC2 are referred to as late‐stage‐enriched and early‐stage‐enriched groups, respectively.

The 10 gene co‐expression modules were annotated by ORA and PPI analysis using Metascape, yielding the following module designations (Figure 1C; Tables S2A and S3B): M1, Inflammatory Myeloid/Cytokine Activation; M2, Nucleosome/Chromatin Remodeling and IFN‐γ/MHC Antigen Presentation; M3, Melanocytic Differentiation Program; M4, Ribosomal Translation and Mitochondrial OXPHOS; M5, Neurodevelopmental/Skeletal Morphogenesis; M6, Keratinocyte Differentiation and Cornified Envelope Formation; M7, Fibroblast‐associated ECM/Matrisome Program; M8, Epidermal Differentiation, Adhesion and IFN/Chemokine Signaling; M9, MITF‐Melanocytic and Collagen/ECM‐Stromal Program; and M10, Neuroactive Synaptic Signaling. Comparison of the module scores between HC1 and HC2 identified six modules with significantly different scores (Figure 1D; Figure S2A); M1, M2, M9, and M10 were enriched in HC1, whereas M6 and M8 were enriched in HC2. Among the six modules that differed significantly between HC1 and HC2, M10 was the only one in which the rank‐based and mean‐based comparisons disagreed in direction (rank‐biserial *r* = +0.457 favoring HC1, mean difference −0.149; Table S2N), suggesting sensitivity to extreme values. We therefore describe M10 but do not use it to support any conclusion in this study. The robustness of the module definitions to the dendrogram cut, the number of variable genes, and resampling of cases is summarized in Table S2G–M. A direct comparison of late‐ versus early‐stage tumors yielded largely concordant results (Figure S2B; Table S2N): M2 and M3 were higher and M6 and M8 lower in late‐stage tumors after BH correction, whereas the differences in M1, M9, and M10 were not significant. GSEA of the HALLMARK gene sets also identified differences in biological programs between HC1 and HC2 (Figure 1E; Table S3A): HC2‐positive programs included tissue architecture maintenance (apical junction, NOTCH signaling) and p53 pathway activation, whereas HC1‐positive programs were dominated by cell‐cycle activation and anabolic growth signatures. In preranked analyses under the four limma models, 17 of the 25 HALLMARK gene sets that were significant between late‐ and early‐stage tumors in the primary model remained significant in the same direction after adjustment for histological tumor purity (Table S3G,H).

### 3.2 A Module Co‐Expression Network Places M9 and M1 at Its Center

To examine inter‐module relationships, we constructed a co‐expression network in which nodes represent modules and edges represent BH‐significant pairwise Spearman correlations (26 correlations, of which only M6–M10 was negative) (Figure 2A; full pairwise correlation matrix shown in Figure S3; Table S2B). The HC1‐associated modules M1, M2, and M9, together with the melanocytic module M3, whose mean score was higher in HC1 but whose HC1–HC2 comparison was not significant after BH correction, formed a densely inter‐correlated block. M9 (MITF‐Melanocytic and Collagen/ECM‐Stromal Program) was strongly correlated with M1 (Spearman *ρ* = 0.90) and M3 (*ρ* = 0.78), and M9 and M1 had the two highest weighted eigenvector centralities in the network (1.000 and 0.997; Figure 2B). M9 and M1 remained the two most central modules when M10, the module most strongly correlated with M9 (*ρ* = 0.91), was excluded from the network. Figure 2C summarizes the module differences between HC1 and HC2 tumors.

**FIGURE 2:**
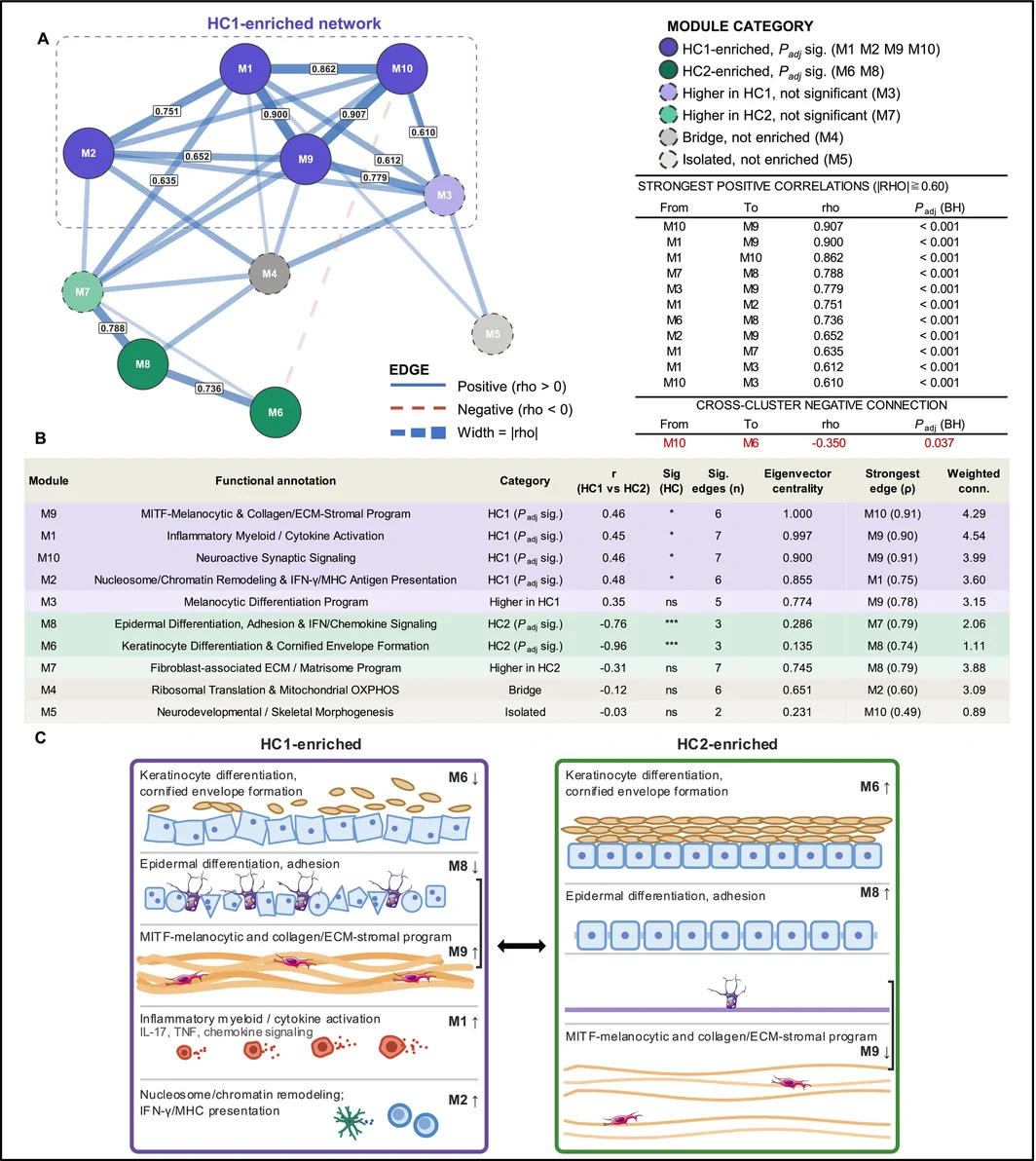
A module co‐expression network of HC1 (late)‐enriched and HC2 (early)‐enriched modules. (A) Module co‐expression network. Nodes are modules M1–M10; edges are pairwise Spearman correlations of module scores across the 43 tumors with BH‐adjusted *p* < 0.05 (BH across the 45 module pairs; Table S2B); solid blue edges, positive correlations (width proportional to |*ρ*|); dashed red edge, the only significant negative correlation (M6–M10). Edge labels give *ρ* for pairs with *ρ* ≥ 0.60. Nodes are colored by category: HC1‐enriched (*p*_adj_ < 0.05; M1, M2, M9, M10); HC2‐enriched (*p*_adj_ < 0.05; M6, M8); higher in HC1 or HC2, not significant—the two nonsignificant modules with the largest absolute rank‐biserial *r* (M3, *r* = 0.35; M7, *r* = −0.31). Among the remaining nonsignificant modules, M4 is labeled Bridge (six significant edges) and M5 Isolated (two significant edges). M9 and M1 had the two highest eigenvector centralities. Right: Module pairs with *ρ* ≥ 0.60 and the negative correlation, with BH‐adjusted *p*. (B) Module summary. *r*, rank‐biserial correlation (HC1 vs. HC2); Sig (HC), significance symbols based on *p*_adj_ of the HC1–HC2 comparison (two‐sided exact Wilcoxon rank‐sum test, BH across 10 modules); Sig. edges (*n*), number of significant correlations involving the module, irrespective of sign; eigenvector centrality, *ρ*‐weighted eigenvector centrality in the network of significant positive edges, rescaled to a maximum of 1; strongest edge, the partner module and ρ of the strongest significant positive edge; weighted conn., sum of ρ over the significant positive edges (Table S2A). (C) Schematic summary of the module differences between HC1 and HC2 tumors. HC1 tumors had higher scores of the MITF‐melanocytic and collagen/ECM‐stromal module (M9), inflammatory myeloid/cytokine activation (M1), and nucleosome/chromatin remodeling and IFN‐γ/MHC antigen presentation (M2), and HC2 tumors had higher scores of keratinocyte differentiation (M6) and epidermal differentiation/adhesion (M8). Arrows indicate higher (↑) or lower (↓) module scores relative to the other group (*p*_adj_ < 0.05; Figure 1D). M10, which was also higher in HC1, is not shown because it is not used to support any conclusion (Section 3). Asterisks denote *p*_adj_ (*, < 0.05; **, < 0.01; ***, < 0.001); ns, not significant. HC1, *n* = 17; HC2, *n* = 26.

To assess whether the network merely re‐expresses a single dominant axis of variation, we recomputed the inter‐module correlations after conditioning on the first principal component of the expression data (Section 2). Of the 27 module pairs with |*ρ*| ≥ 0.3 in the unconditioned network, 18 retained |*ρ*| ≥ 0.3 with the same sign, including the strongest edges; the edges that did not persist were concentrated on M7 and M4 (Table S2P). After conditioning on the HC partition, 25 of the 27 pairs retained |*ρ*| ≥ 0.3 with the same sign. M9 and M1 remained the two most central modules after conditioning on the first principal component (Table S2Q).

### 3.3 Functional Enrichment and PPI Analyses Identify Collagen/ECM‐Related Components Within M9

We characterized the functional enrichment and PPI structure of M9 (Figure 3). Metascape ORA revealed that the top‐ranked pathways encompassed *MITF*‐M‐regulated melanocyte development (R‐HSA‐9730414) and collagen formation (R‐HSA‐1474290), alongside cytokine signaling, cell‐motility regulation, and *MITF*‐dependent gene programs governing DNA replication and senescence (Figure 3A). PPI network analysis identified nine densely connected components by MCODE (Figure 3B,C; Table S3D–F). MCODE1 contained collagen‐chain and collagen‐processing genes, including *COL11A2*, *COL9A3*, *COL22A1*, and *PLOD3*.

**FIGURE 3:**
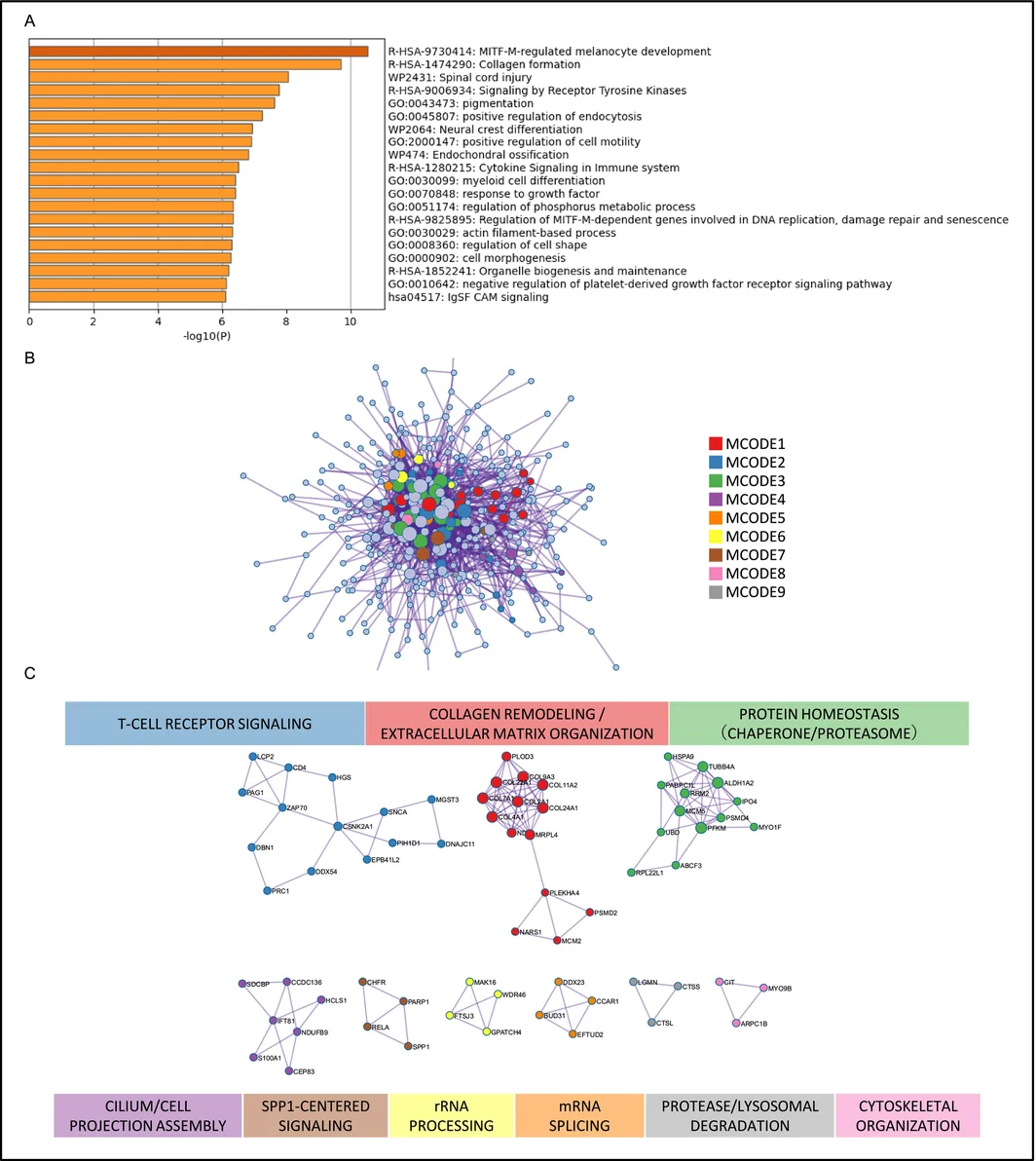
Functional enrichment and protein–protein interaction (PPI) analysis of the M9 MITF‐Melanocytic and Collagen/ECM‐Stromal Program module. (A–C) show the results for the 404 genes of module M9. (A) The 20 most enriched terms (Metascape over‐representation analysis; one representative term per cluster of redundant terms), ranked by −log10(*p*); bar lengths represent nominal enrichment *p* values, and Metascape *q* values are reported in Table S3B. (B) PPI network of the M9 genes built in Metascape (333 proteins, 1129 interactions). Densely connected components identified by MCODE are colored (MCODE1–9; 66 proteins). MCODE numbers follow the Metascape output (Table S3D–F). (C) MCODE components grouped by function. Functional labels are author‐assigned descriptions based on component gene membership and, where available, enrichment results (Table S3D,E). MCODE1 (red) contained collagen‐chain and collagen‐processing genes, providing a collagen/extracellular matrix (ECM)‐related annotation for this PPI component.

### 3.4 Collagen/ECM Gene Expression Differs Between HC1 (Late‐Stage‐Enriched) and HC2 (Early‐Stage‐Enriched) Tumors

We examined genome‐wide differential expression results for the 91‐gene collagen/ECM compilation (Section 2; Figure 4A; Table S4A). Of the 91 genes, 59 and 56 had genome‐wide *p*_adj_ < 0.05 in the HC1‐versus‐HC2 and late‐versus‐early‐stage comparisons, respectively. After additional adjustment for histological tumor purity, 8 genes met this criterion in the HC comparison and only *PLOD3* did so in the stage comparison (Table S4A). Of the 91 genes, 35 met the HC1‐versus‐HC2 criteria (genome‐wide *p*_adj_ < 0.05 and |log_2_ fold change| ≥ 1) and changed in the same direction in late‐ versus early‐stage tumors: 18 were higher in HC1 and 17 in HC2 (Table S4B). These genes were annotated to intracellular collagen processing, ECM assembly, and ECM remodeling (Figure 4B). Figure 4C illustrates *PLOD3*, *COL11A1*, and *MMP1* as examples from these respective categories. *COL17A1*, *SERPINB7*, *LAMA3*, and *TGFB3* were among the genes expressed at lower levels in HC1 (Table S4B; Figures S4 and S5). Genes involved in collagen synthesis (*COL11A1*/*COL11A2*), cross‐linking (*LOXL2*), and proteolytic remodeling (*MMP1*/*MMP9*/*CTSL*) were more highly expressed in HC1 (Table S4A). None of the externally defined collagen gene‐set scores, including versions without the genes shared with the 14‐gene score (Methods), differed significantly between late‐ and early‐stage tumors (Table S4C).

**FIGURE 4:**
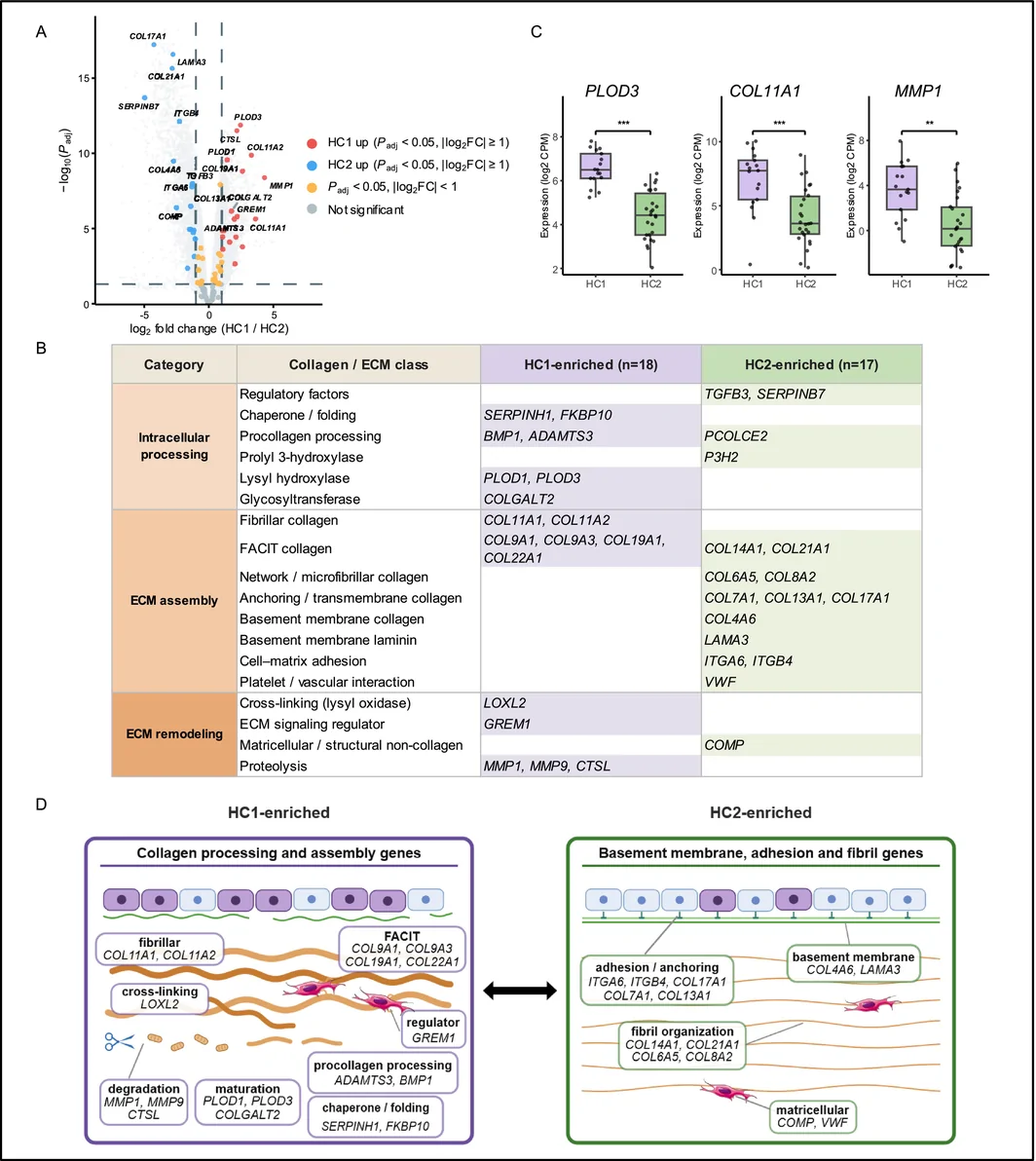
Collagen/ECM gene expression differences between HC1 (late‐enriched) and HC2 (early‐enriched) tumors. (A) Volcano plot of the 91 collagen/ECM genes examined (Table S4A) for HC1 versus HC2 (limma–voom primary model, expression ~ HC group + age + sequencing batch; BH correction across all 17,485 genes). Red, higher in HC1; blue, higher in HC2 (*p*_adj_ < 0.05 and |log_2_ fold change| ≥ 1); orange, *p*_adj_ < 0.05 with |log_2_ fold change| < 1; gray, not significant. (B) The 35 collagen/ECM genes meeting the HC1‐versus‐HC2 criteria of *p*_adj_ < 0.05 and |log_2_ fold change| ≥ 1, with concordant directions of change in late‐ versus early‐stage tumors (18 higher in HC1 and 17 higher in HC2), grouped by collagen/ECM class and by step (intracellular processing, ECM assembly, ECM remodeling) (Table S4B). FACIT, fibril‐associated collagens with interrupted triple helices. (C) Expression (log_2_ CPM) of *PLOD3*, *COL11A1*, and *MMP1* in HC1 versus HC2, as examples of the intracellular processing, ECM assembly, and ECM remodeling steps, respectively. Two‐sided Wilcoxon rank‐sum test with BH correction across the 35 genes in (B) (Table S4B). (D) Schematic summary of the genes in (B), arranged by collagen/ECM step for HC1 (late‐enriched, left) and HC2 (early‐enriched, right) tumors. The schematic reflects gene expression inferred from RNA‐seq and does not represent histological or biochemical measurements. Asterisks denote *p*_adj_ (*, < 0.05; **, < 0.01; ***, < 0.001). HC1, *n* = 17; HC2, *n* = 26.

### 3.5 Immune and Stromal Features of HC1 and HC2 Tumors and Associations of Macrophage Signature Scores With Collagen Biosynthesis Gene Expression

To characterize the immune and stromal features of HC1 and HC2 tumors, we applied TIMER3 deconvolution to the 43 tumor specimens (Figure 5A; Table S5A,B). The cell types that met the concordance criterion (a significant difference in the same direction in two or more algorithms) were macrophages (3 of the 5 algorithms providing an estimate), M1‐labeled macrophages (2 of 4), M2‐labeled macrophages (2 of 4), and B cells (3 of 7), all higher in HC1, and CD8+ T cells (3 of 8) and the stroma score (2 of 2), higher in HC2. In the late‐ versus early‐stage comparison, no cell type met the concordance criterion (Figure S6; Table S5B).

**FIGURE 5:**
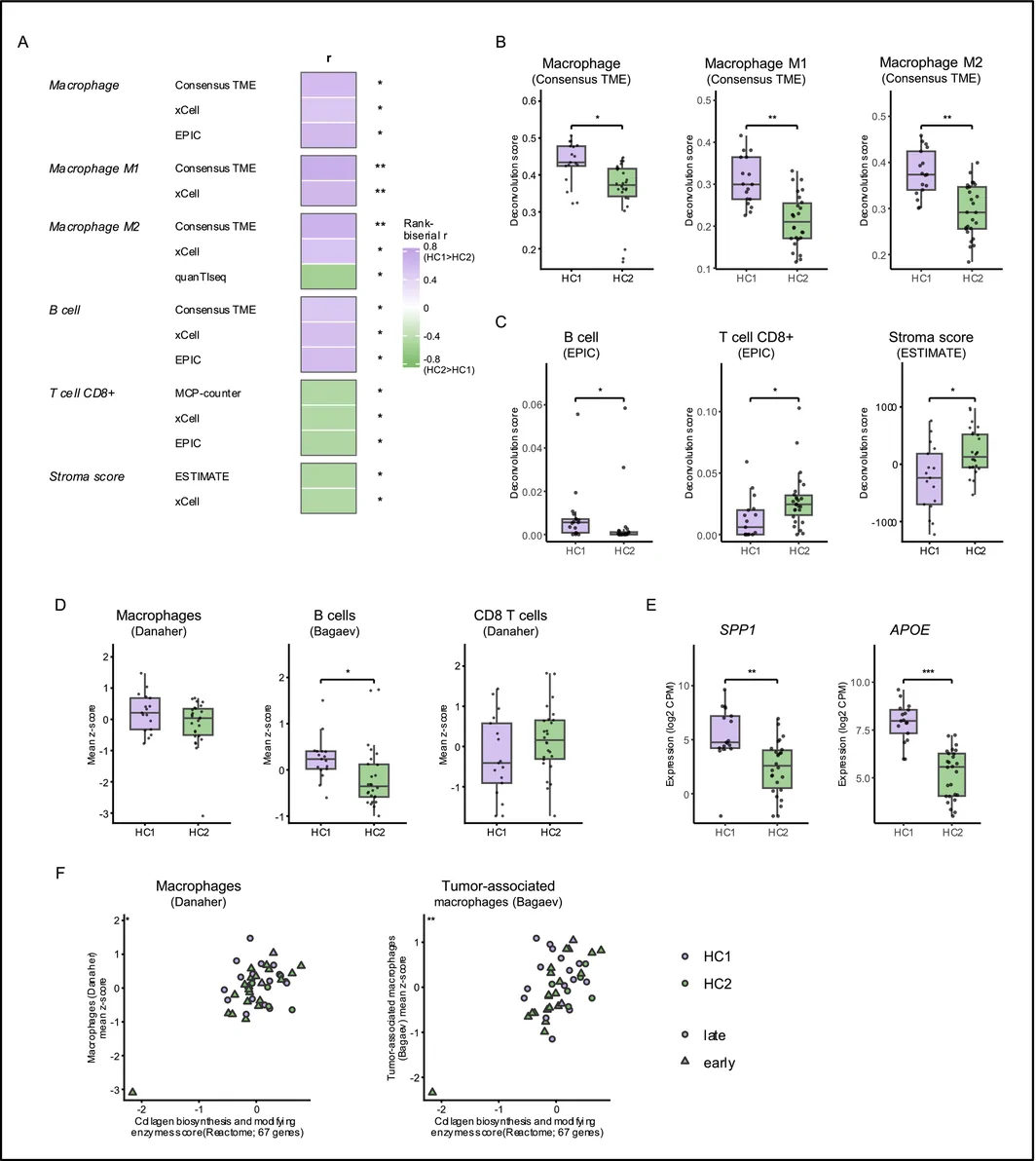
Immune and stromal cell estimates and immune‐related gene expression in HC1 versus HC2 tumors, and associations of macrophage signature scores with a collagen biosynthesis gene‐set score. (A) Rank‐biserial *r* for HC1 versus HC2 of TIMER3 cell‐type estimates. Shown are the cell types defined as consistently altered (significant in the same direction in two or more algorithms; Table S5B), with one row for each algorithm with *p*_adj_ < 0.05. Relative CIBERSORT estimates were not counted in these numbers (they remain in the BH correction across all 182 estimates). Numbers of concordant significant algorithms/algorithms providing an estimate: Macrophages, 3/5; M1‐labeled macrophages, 2/4; M2‐labeled macrophages, 2/4 (quanTIseq, significant in the opposite direction); B cells, 3/7; CD8+ T cells, 3/8; stroma score, 2/2. Purple, higher in HC1; green, higher in HC2. The ESTIMATE and xCell stroma scores are composite scores of nonhematopoietic stromal signals and do not distinguish fibroblasts, endothelial cells, and pericytes (fibroblast and endothelial cell estimates, Figure S7). (B) Macrophage, M1‐labeled and M2‐labeled macrophage estimates (ConsensusTME). (C) B cell and CD8+ T cell estimates (EPIC) and the stroma score (ESTIMATE). (D) Scores of externally defined gene signatures (mean gene‐wise z‐score of log_2_ counts per million (CPM) across all 43 tumors): Macrophages (Danaher et al. 2017), B cells (Bagaev et al. 2021), and CD8 T cells (Danaher et al. 2017) (definitions, Table S6A). The tumor‐associated macrophage signature (Bagaev et al. 2021) is shown in Figure S8A. (E) Expression (log_2_ CPM) of *SPP1* and *APOE*. (F) Spearman correlation across all 43 tumors between the collagen biosynthesis and modifying enzymes gene‐set score (REACTOME; 67 genes; Table S6A) and the macrophage (Danaher et al. 2017) and tumor‐associated macrophage (Bagaev et al. 2021) signature scores. Point color (HC group) and shape (stage) are for display only. Asterisks denote *p*_adj_ (BH across four correlations). *ρ*, 95% bootstrap confidence intervals (not adjusted for multiplicity) and purity‐adjusted partial correlations are given in Table S6C. (A–E): two‐sided Wilcoxon rank‐sum test (HC1 vs. HC2) with BH correction within each family: (A–C), all 182 TIMER3 estimates (Table S5A); (D), the four signatures (Table S6B); (E), the 24 macrophage‐associated genes (Table S6B,D). Asterisks denote *p*_adj_ (*, < 0.05; **, < 0.01; ***, < 0.001). HC1, *n* = 17; HC2, *n* = 26.

Macrophage, M1‐labeled, and M2‐labeled macrophage estimates from ConsensusTME were higher in HC1 (Figure 5B). B cell estimates were higher and CD8+ T cell estimates lower in HC1 (EPIC), and the ESTIMATE stroma score was lower in HC1 (Figure 5C). Among externally defined gene signatures, the B cell signature (Bagaev et al. 2021) was higher in HC1 (*r* = 0.48, *p*_adj_ = 0.035), whereas the macrophage and CD8 T cell signatures (Danaher et al. 2017) differed in the same directions as the deconvolution estimates but did not reach the BH criterion (*p*_adj_ = 0.176 for both; Figure 5D; Table S6B). To identify the cell types underlying the stroma score difference, we examined fibroblast and endothelial cell estimates from four algorithms (Figure S7; Table S5A). Fibroblast estimates did not differ significantly between the groups in any algorithm and did not agree in direction; endothelial cell estimates were lower in HC1 in three of the four algorithms, but the difference was significant only for EPIC. The cell types underlying the stroma score difference could therefore not be identified. Across all tumors, the MCP‐counter fibroblast estimate was correlated with a Reactome collagen biosynthesis gene‐set score from which its marker genes had been removed (*ρ* = 0.70, 95% confidence interval 0.44–0.87; Table S5E); this score is derived from bulk expression and does not identify the cells producing collagen.

Among the 24 macrophage‐associated genes examined (Section 2), *APOE* and *SPP1* showed the largest differences between HC1 and HC2 and between late‐ and early‐stage tumors; both were more highly expressed in HC1 (Figure 5E) and in late‐stage tumors (Figure S6E; Table S6B,D). The tumor‐associated macrophage signature score (Bagaev et al. 2021) did not differ significantly between HC1 and HC2 (Figure S8A; Table S6B). Across all 43 tumors, the macrophage signature score (Danaher et al. 2017) and the tumor‐associated macrophage signature score were positively correlated with the Reactome collagen biosynthesis and modifying enzymes gene‐set score (*ρ* = 0.39, 95% confidence interval 0.05–0.64, *p*_adj_ = 0.019; and *ρ* = 0.47, 0.18–0.69, *p*_adj_ = 0.006, respectively), and the correlations remained significant after adjustment for histological tumor purity (partial *ρ* = 0.41, *p*_adj_ = 0.013; and 0.49, *p*_adj_ = 0.004) (Figure 5F; Table S6C).

To examine T‐cell states beyond CD8+ T‐cell abundance, we compared an activated CD8 T‐cell signature and an inhibitory receptor signature between the groups (Figure S8B–D; Table S6F,G). Neither signature score differed significantly between HC1 and HC2 or between late‐ and early‐stage tumors after BH correction, either without adjustment or after adjustment for the MCP‐counter CD8+ T‐cell estimate.

Histological tumor purity, estimated within the macrodissected tumor area (Figure S9), was correlated with stage (Section 2), and tissue composition may contribute to the collagen‐ and macrophage‐associated expression differences. In sensitivity analyses additionally adjusted for histological tumor purity, the HC and stage coefficients of *COL11A1*, *BMP1*, *SPP1*, and *APOE* were attenuated, and none remained significant after BH correction (Table S7A). With AJCC T category as an alternative progression axis, *APOE* remained significantly associated after purity adjustment (Table S7B). Because purity adjustment can also remove stage‐associated signal, these results indicate that the stage‐associated differences could not be separated from tissue composition in this dataset; they do not show that tissue composition explains these differences. The overall correlation between histological and ESTIMATE‐derived purity was weak (*ρ* = 0.15, 95% confidence interval −0.21 to 0.48), with positive and negative correlations in late‐ and early‐stage tumors, respectively (Table S7C,D).

### 3.6 Exploratory Cross‐Subtype Comparison of Collagen/ECM Gene Expression and Immune Features Across Nail, Plantar, and Nonacral Melanomas

We explored differences in collagen/ECM gene expression and immune features between late‐stage nail unit melanoma (*n* = 21) and 12 late‐stage nonnail melanomas (plantar acral, *n* = 6; nonacral cutaneous, *n* = 6) (Table S8A). None of the collagen/ECM genes, collagen scores, or immune cell estimates differed significantly between nail unit and nonnail melanoma after BH correction (Figure S10; Table S8B,D). In the three‐group comparison, only *COL19A1* showed a significant Kruskal–Wallis test after BH correction (*p*_adj_ = 0.009), with all three pairwise Dunn comparisons significant (Table S8C); the genes and immune estimates shown in Figure 6 did not meet the Kruskal–Wallis criterion (Figure 6; Table S8C,E). These exploratory findings require evaluation in larger cohorts.

**FIGURE 6:**
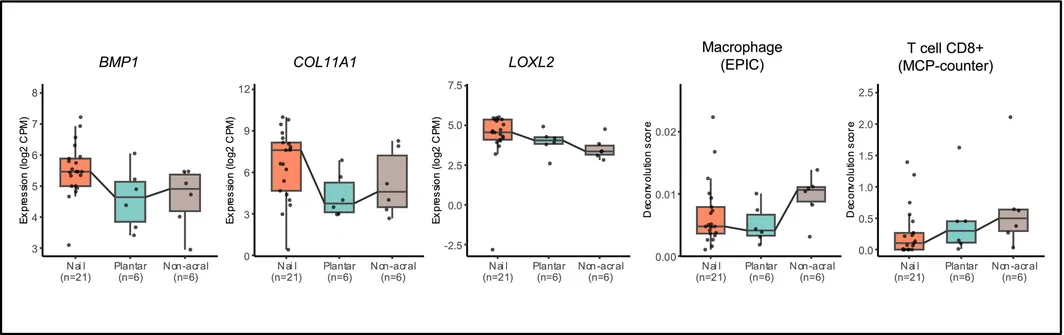
Exploratory comparison of collagen/ECM genes and immune cell estimates across late‐stage nail unit, plantar acral, and nonacral melanoma. Expression (log_2_ CPM) of *BMP1*, *COL11A1* and *LOXL2*, and macrophage (EPIC) and CD8+ T cell (MCP‐counter) estimates in late‐stage (Stage IIB or higher) nail unit melanoma (*n* = 21), plantar acral melanoma (*n* = 6) and nonacral melanoma (*n* = 6). Lines connect group medians. Kruskal–Wallis test with BH correction across the 37 genes and scores or the 12 cell‐type estimates, with pairwise Dunn tests (BH correction across the three pairwise comparisons) interpreted only for genes, scores or cell‐type estimates with a significant Kruskal–Wallis test (*p*_adj_ < 0.05) (Table S8C,E). None of the five displayed features met the BH‐adjusted Kruskal–Wallis criterion for post hoc interpretation; Dunn results for all pairs are given in Table S8C,E. The analysis is exploratory; the comparison of nail unit versus nonnail melanoma is shown in Figure S10. CPM, counts per million.

Within the nail unit cohort, late‐stage lesions were more frequent among toenail than fingernail lesions (9 of 14 vs. 12 of 29), although the difference was not significant (Fisher's exact *p* = 0.203; Table S9A). After adjustment for lesion category, age, and sequencing batch, none of the collagen, module, or immune scores shown in Table S9B differed significantly between toenail and fingernail lesions, with or without adjustment for histological tumor purity. With only 14 toenail lesions, these estimates were imprecise.

## 4 Discussion

In this study, bulk transcriptomic profiling of 43 nail unit melanomas identified two transcriptomic groups associated with clinical stage. The late‐stage‐enriched group (HC1) was characterized by higher expression of genes involved in collagen synthesis, cross‐linking, and remodeling, higher macrophage and lower CD8+ T‐cell estimates, and higher scores of a module combining melanocytic and collagen/ECM‐stromal genes (M9), which, together with the inflammatory myeloid module (M1), occupied the most central position in the inter‐module co‐expression network. Across tumors, macrophage‐associated signature scores were correlated with a collagen biosynthesis gene‐set score, including after adjustment for histological tumor purity. These findings indicate that stromal and myeloid features co‐vary in nail unit melanoma.

Both HC1 and HC2 tumors expressed collagen/ECM genes, but different ones: of the 35 genes that met the HC1‐versus‐HC2 criteria and showed concordant directions of change in the stage comparison, 18 were higher in HC1 and 17 in HC2. Genes higher in HC1 and in late‐stage tumors included *COL11A1*, *LOXL2*, and *PLOD3*, which act in collagen assembly, cross‐linking, and processing, whereas genes higher in HC2 included the hemidesmosome and basement‐membrane genes *COL17A1* and *LAMA3*. *COL11A1* is part of an invasion‐associated desmoplastic expression signature shared across cancers (Kim et al. 2010) and is expressed by cancer‐associated fibroblasts (Zhu et al. 2021). In acral melanoma, ECM organization pathways were enriched in invasive compared with in situ tumors (Liu et al. 2024). M9 combined melanocytic and collagen/ECM genes, and its PPI network contained a collagen‐processing component, highlighting the coordinated expression of these features at the tissue level. Although the Reactome collagen biosynthesis gene‐set score did not differ significantly between early‐ and late‐stage tumors, several individual collagen/ECM genes showed stage‐associated differences.

The deconvolution estimates suggested differences in the immune profile between HC1 and HC2, with higher macrophage and B‐cell estimates but lower CD8+ T‐cell estimates in HC1. The activated CD8+ T‐cell and inhibitory receptor signature scores did not differ significantly between the groups. *SPP1* and *APOE* were more highly expressed in HC1 and in late‐stage tumors. In acral melanoma, infiltration by macrophages characterized by APOE and CD163 expression was associated with shorter progression‐free survival (Liu et al. 2024). In a single‐cell study of primary acral melanoma, *SPP1*+ macrophages, which included *APOE*‐expressing subsets, accounted for a higher proportion of myeloid cells in tumors with lymph node metastasis (Liang et al. 2026). A reanalysis of single‐cell data from melanoma biopsies obtained before or during immune checkpoint therapy identified a *TREM2*‐high macrophage subset expressing *SPP1* and complement genes that was enriched in nonresponders (Xiong et al. 2020). These findings provide a context for the higher *SPP1* and *APOE* expression observed in HC1 and late‐stage tumors, although our bulk transcriptomic data do not establish their cellular sources or immunosuppressive functions. Whether *SPP1* and *APOE* expression is associated with immune checkpoint inhibitor response in nail unit melanoma requires investigation in treated cohorts.

Across all tumors, macrophage‐associated signature scores were positively correlated with the Reactome collagen biosynthesis gene‐set score, and these correlations persisted after adjustment for histological tumor purity. Experimental work in breast cancer has linked a stiff fibrotic microenvironment to macrophage collagen production and metabolic changes that impair CD8+ T cell function (Tharp et al. 2024). Our observations are compatible with this model and suggest that collagen/ECM remodeling and macrophage‐associated programs may be linked in nail unit melanoma, a hypothesis that can be tested with spatial and functional approaches. Whether these collagen/ECM‐ and macrophage‐associated expression features contribute to the non–T cell‐inflamed phenotype and the limited efficacy of immune checkpoint inhibitors reported in acral melanoma (Augustin et al. 2023; Nakamura et al. 2020) remains to be determined. In a murine pancreatic cancer model, inhibition of collagen cross‐linking enhanced the response to anti‐PD‐1 treatment (Nicolas‐Boluda et al. 2021). SPP1 neutralization reduced tumor growth in a murine melanoma model (Liang et al. 2026), while TREM2‐targeting antibodies enhanced anti‐PD‐1 activity in a murine sarcoma model (Molgora et al. 2020).

Some features of nail unit melanoma may be associated with its anatomical site, which has specialized dermal and mesenchymal compartments. A prior single‐cell study reported higher *COL11A1* expression among malignant‐cell‐associated differentially expressed genes in subungual than in plantar melanoma (Wang et al. 2024). In our exploratory cross‐subtype comparison, *COL11A1* expression was numerically higher in nail unit melanoma than in the pooled plantar and nonacral cutaneous melanoma group, although this difference was not significant after BH correction. Within the nail unit cohort, none of the analyzed scores differed significantly between fingernail and toenail lesions.

This study had several limitations. First, our conclusions are derived from bulk transcriptomic data, which cannot resolve the cellular source of individual transcripts; the inferred macrophage‐, stromal‐, and lymphocyte‐associated programs will require validation by single‐cell or spatial methods and by protein‐level assays. Second, immune cell estimates were obtained by computational deconvolution, which provides relative rather than absolute quantification; because bulk deconvolution and tumor‐purity estimates capture tissue composition only coarsely, residual differences in stromal and immune content may still contribute to the observed signatures. Third, the stage‐associated differences in collagen‐ and macrophage‐related genes were attenuated after adjustment for histological tumor purity; because purity was correlated with stage, the contributions of tissue composition and disease stage could not be separated. Fourth, HC1 and HC2 were defined from the same expression data that were compared between them, so these differences are descriptive. Fifth, the cross‐subtype comparison with plantar and nonacral melanoma was based on a small additional cohort and requires confirmation in larger, independent cohorts. Finally, the study design was retrospective and cross‐sectional, so that the stage‐associated differences reflect comparisons between tumors from different patients rather than longitudinal sampling, and direct associations with the response to immunotherapy could not be assessed.

In conclusion, this study describes stage‐associated differences in collagen/ECM gene expression and co‐variation of macrophage‐associated and collagen biosynthesis programs in nail unit melanoma. These features provide a basis for single‐cell, spatial, and functional studies of the stromal–myeloid microenvironment of this rare melanoma subtype and for evaluating its relevance to the response to immunotherapy.

## Author Contributions

**Yuri Sawada:** writing – original draft, writing – review and editing, conceptualization, methodology, software, data curation, investigation, validation, formal analysis, visualization, resources. **Marco A. De Velasco:** writing – review and editing, conceptualization, methodology, software, investigation, validation, formal analysis, visualization. **Megumi Aoki:** writing – review and editing, data curation, resources. **Kazuko Sakai:** writing – review and editing, conceptualization, methodology, investigation. **Naoki Shiraishi:** writing – review and editing, conceptualization, investigation. **Kazuyasu Fujii:** writing – review and editing, conceptualization, resources. **Shigeto Matsushita:** writing – review and editing, conceptualization, resources. **Kazuto Nishio:** writing – review and editing, conceptualization, methodology, supervision, project administration. **Atsushi Otsuka:** writing – review and editing, conceptualization, resources, supervision.

## Funding

The authors have nothing to report.

## Ethics Statement

This study was reviewed and approved by the Ethics Committee of the Faculty of Medicine, Kindai University, and was conducted with the implementation authorization of the Dean of the Faculty of Medicine (granted 22 May 2024). The use and provision of archival specimens at participating institutions were conducted in accordance with the applicable institutional ethical procedures. All procedures were performed in accordance with the Declaration of Helsinki.

## Consent

Written informed consent was obtained from prospectively enrolled participants. For archival cases, the requirement for written informed consent was waived under opt‐out procedures approved in accordance with the ethical requirements of the relevant participating institutions, whereby information about the study was made publicly available and patients were given the opportunity to decline the use of their samples and data; specimens from patients who declined were excluded.

## Conflicts of Interest

K.S. has received speaker's fees from Qiagen, Life Technologies Japan, and Chugai Pharmaceutical. S.M. has received speaker's fees from Ono Pharmaceutical, Novartis, Bristol‐Myers Squibb, and Merck Sharp & Dohme. A.O. has received speaker's fees from Eli Lilly Japan K.K. and AbbVie GK. The other authors declare no conflicts of interest.

## Supporting information


**Figure S1:** Louvain clustering and quality and stability of the hierarchical clustering. (A) t‐SNE of the 43 tumors based on the 2000 most variable genes, colored by Louvain cluster (C1–C4; Orange3); symbols indicate stage (early, circles; late, crosses). C4 contains a single sample. The colored background regions are drawn by the Orange3 scatter‐plot display and are shown for C1–C3; they are not an analytical result. Correspondence with HC1/HC2 and adjusted Rand indices are given in Table S2D. (B) Mean silhouette width of the sample clustering for *k* = 2–5; values are shown above the points. (C) Silhouette width of each tumor at *k* = 2, ordered within HC1 (*n* = 17) and HC2 (*n* = 26). (D) Bootstrap stability of HC1 and HC2 (mean clusterwise Jaccard coefficient over 1000 case resamples) with the 2000 genes fixed (Fixed) or with the 2000 most variable genes re‐selected in each resample (Re‐selected). Dashed reference lines mark Jaccard coefficients of 0.75 and 0.60. In (B)–(D), samples were clustered by 1 − Pearson correlation of gene‐wise z‐scored expression with complete linkage (Table S2E,F).

**Figure S2:** Module scores in HC and stage comparisons. (A) Module scores (mean gene‐wise *z*‐score) of M3, M4, M5, and M7 in HC1 (*n* = 17) versus HC2 (*n* = 26). (B) Module scores of M1–M10 in late‐stage (*n* = 21) versus early‐stage (*n* = 22) tumors. Two‐sided Wilcoxon rank‐sum test (exact) with BH correction across the 10 modules within each comparison; asterisks denote *p*_adj_ (*, < 0.05; **, < 0.01; ***, < 0.001); no marker, *p*_adj_ ≥ 0.05 (Table S2N).

**Figure S3:** Pairwise correlations among the 10 module scores. Spearman correlation coefficients (*ρ*) between module scores across the 43 tumors; modules are ordered by hierarchical clustering. Asterisks mark pairs with BH‐adjusted *p* < 0.05 (BH across the 45 pairs; Table S2B).

**Figure S4:** Expression of the 35 differentially expressed collagen/ECM genes in HC1 versus HC2. Expression (log_2_ CPM) of the genes in Figure 4B (Table S4B) in HC1 (*n* = 17) versus HC2 (*n* = 26). Two‐sided Wilcoxon rank‐sum test with BH correction across the 35 genes. Asterisks denote BH‐adjusted *p*: *, < 0.05; **, < 0.01; ***, < 0.001; no asterisk, ≥ 0.05.

**Figure S5:** Expression of the 35 collagen/ECM genes in late‐ versus early‐stage tumors. Expression (log_2_ CPM) of the genes in Figure 4B (Table S4B) in late‐stage (*n* = 21) versus early‐stage (*n* = 22) tumors. Statistical tests and symbols are as in Figure S4.

**Figure S6:** Immune and stromal cell estimates and immune‐related gene expression in late‐ versus early‐stage tumors. Late‐stage (*n* = 21) versus early‐stage (*n* = 22) comparisons corresponding to Figure 5A–E. (A) Rank‐biserial *r* (late vs. early) of TIMER3 estimates. Because no cell type met the two‐algorithm criterion on the stage axis (Table S5B), the panel shows, among the cell types shown in Figure 5A, the estimates with *p*_adj_ < 0.05 on the stage axis (macrophages, M1‐labeled and M2‐labeled macrophages; ConsensusTME; relative CIBERSORT estimates not shown). (B) Macrophage, M1‐labeled and M2‐labeled macrophage estimates (ConsensusTME). (C) B cell and CD8+ T cell estimates (EPIC) and the stroma score (ESTIMATE). (D) Macrophages (Danaher et al. 2017), B cells (Bagaev et al. 2021) and CD8 T cells (Danaher et al. 2017) signature scores. (E) *SPP1* and *APOE* expression (log_2_ CPM). Two‐sided Wilcoxon rank‐sum test. BH correction was applied separately on the stage axis across all 182 TIMER3 estimates for (A)–(C), the four gene signatures for (D) (including the tumor‐associated macrophage signature in Figure S8A), and the 24 macrophage‐associated genes for (E) (Tables S5A and S6B,D). Asterisks denote *p*_adj_ (*, < 0.05; **, < 0.01; ***, < 0.001). Orange, late; blue, early.

**Figure S7:** Fibroblast and endothelial cell estimates. Fibroblast and endothelial cell estimates from four TIMER3 algorithms (xCell, EPIC, ConsensusTME, MCP‐counter) in (A) HC1 (*n* = 17) versus HC2 (*n* = 26) and (B) late‐stage (*n* = 21) versus early‐stage (*n* = 22) tumors. Two‐sided Wilcoxon rank‐sum test with BH correction across all 182 TIMER3 estimates within each comparison (Table S5A); asterisks denote *p*_adj_ < 0.05. The xCell fibroblast estimate was below 10^−6^ in 42 of 43 samples.

**Figure S8:** Tumor‐associated macrophage and T‐cell signatures. (A) Score of an externally defined tumor‐associated macrophage gene signature (Bagaev et al. 2021) in HC1 versus HC2 and late versus early tumors (BH across the four signatures of Figure 5D and this panel, separately for each comparison; Table S6B). (B, C) Activated CD8 T‐cell signature (ACT26; 26 genes) and inhibitory receptor signature (IR5; 5 genes) in HC1 versus HC2 (B) and late versus early (C) tumors (definitions, Table S6F). (D) Points show group coefficients (*β*) of ACT26 and IR5 adjusted for the CD8+ T‐cell estimate (score ~ group + log_2_[MCP‐counter CD8+ T‐cell estimate +1]); *β* > 0, higher in HC1 or in late‐stage tumors; bars, 95% confidence intervals not adjusted for multiplicity. (B)–(D): BH correction across the two signatures within each comparison and analysis (Table S6G). Signature scores were calculated as the mean gene‐wise *z*‐score of log_2_ CPM across the 43 tumors. ACT26 uses the published activated CD8 T‐cell gene set; IR5 comprises five inhibitory receptor genes selected in this study from the immunophenoscore source (Table S6F). HC1, *n* = 17; HC2, *n* = 26; late stage, *n* = 21; early stage, *n* = 22. (A)–(C): two‐sided Wilcoxon rank‐sum tests; (D): two‐sided *t*‐tests of the group coefficient in the linear model. None of the comparisons showed a significant group difference after BH correction.

**Figure S9:** Hematoxylin and eosin (HE)‐stained sections of nail unit melanoma. Representative HE‐stained sections of a nail unit melanoma in situ (Tis, stage 0; A–C) and an invasive nail unit melanoma (T4a, Stage IIB; D–F), both arising on the thumb, at low (A, D), intermediate (B, E) and high (C, F) magnification. Blue lines outline the tumor area marked by a pathologist; this area was macrodissected for RNA extraction, and histological tumor purity was estimated within it (20% and 60%, respectively; histological purity assessment, Table S7D). Scale bars, 10 mm (A, D), 500 μm (B, E) and 50 μm (C, F).

**Figure S10:** Collagen/ECM genes and immune cell estimates in late‐stage nail unit versus nonnail melanoma. Expression (log_2_ CPM) of *BMP1*, *COL11A1* and *LOXL2*, and macrophage (EPIC) and CD8+ T cell (MCP‐counter) estimates in late‐stage nail unit melanoma (*n* = 21) versus late‐stage nonnail melanoma (plantar acral and nonacral, *n* = 12). Lines connect group medians. Two‐sided Wilcoxon rank‐sum test with BH correction across the 37 genes and scores or the 12 cell‐type estimates (Table S8B,D); none was significant after correction. The analysis is exploratory.

**Table S1:** Genome‐wide differential expression in nail unit melanoma.

**Table S2:** Co‐expression modules and their robustness in nail unit melanoma.

**Table S3:** Functional enrichment of the HC1/HC2 contrast and of co‐expression modules, and protein–protein interaction components of module M9.

**Table S4:** Collagen and extracellular matrix (ECM) gene expression and collagen gene‐set scores in nail unit melanoma.

**Table S5:** Immune and stromal cell estimates from TIMER3 deconvolution, and fibroblast–collagen association.

**Table S6:** Immune‐related gene expression and associations with the collagen biosynthesis gene‐set score.

**Table S7:** Sensitivity analyses for tumor purity and AJCC T category.

**Table S8:** Exploratory cross‐subtype comparison: collagen/ECM genes and immune deconvolution in late‐stage nail unit versus plantar and nonacral melanoma.

**Table S9:** Differences between fingernail and toenail melanomas.

## Data Availability

The bulk RNA‐sequencing data generated in this study have been deposited in the Japanese Genotype–phenotype Archive (JGA), operated by the National Bioscience Database Center (NBDC) and the DNA Data Bank of Japan (DDBJ), under accession number JGAS000961, and are available under controlled access for academic research use only through the JGA Data Access Committee.
