# Supplementary material for: Stage‐Associated Collagen Gene Expression and Macrophage‐Associated Immune Features in Nail Unit Melanoma
Source: Pigment Cell Melanoma Res. 2026 Nov 11;39(6):e70143. doi: 10.1111/pcmr.70143 (PMC13755139; doi:10.1111/pcmr.70143)
Supplement: Supplementary file 1 [file PCMR-39-0-s001.pdf]

Figure S7

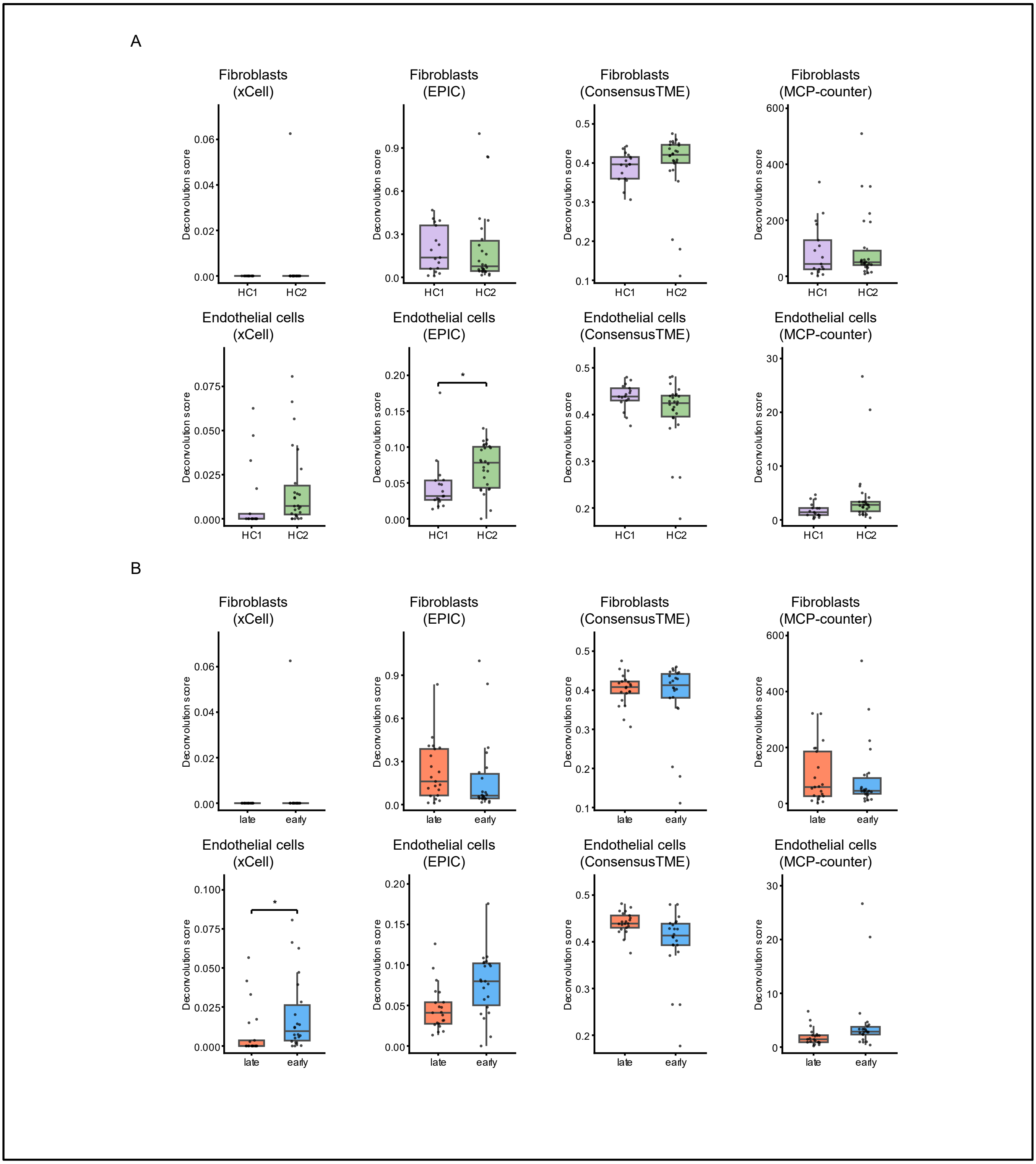

A
Fibroblasts (xCell)
Fibroblasts (EPIC)
Fibroblasts (ConsensusTME)
Fibroblasts (MCP-counter)
Endothelial cells (xCell)
Endothelial cells (EPIC)
Endothelial cells (ConsensusTME)
Endothelial cells (MCP-counter)
Deconvolution score
HC1
HC2
*
B
Fibroblasts (xCell)
Fibroblasts (EPIC)
Fibroblasts (ConsensusTME)
Fibroblasts (MCP-counter)
Endothelial cells (xCell)
Endothelial cells (EPIC)
Endothelial cells (ConsensusTME)
Endothelial cells (MCP-counter)
Deconvolution score
late
early
*
