# Supplementary material for: Stage‐Associated Collagen Gene Expression and Macrophage‐Associated Immune Features in Nail Unit Melanoma
Source: Pigment Cell Melanoma Res. 2026 Nov 11;39(6):e70143. doi: 10.1111/pcmr.70143 (PMC13755139; doi:10.1111/pcmr.70143)
Supplement: Supplementary file 2 [file PCMR-39-0-s002.pdf]

Figure S1

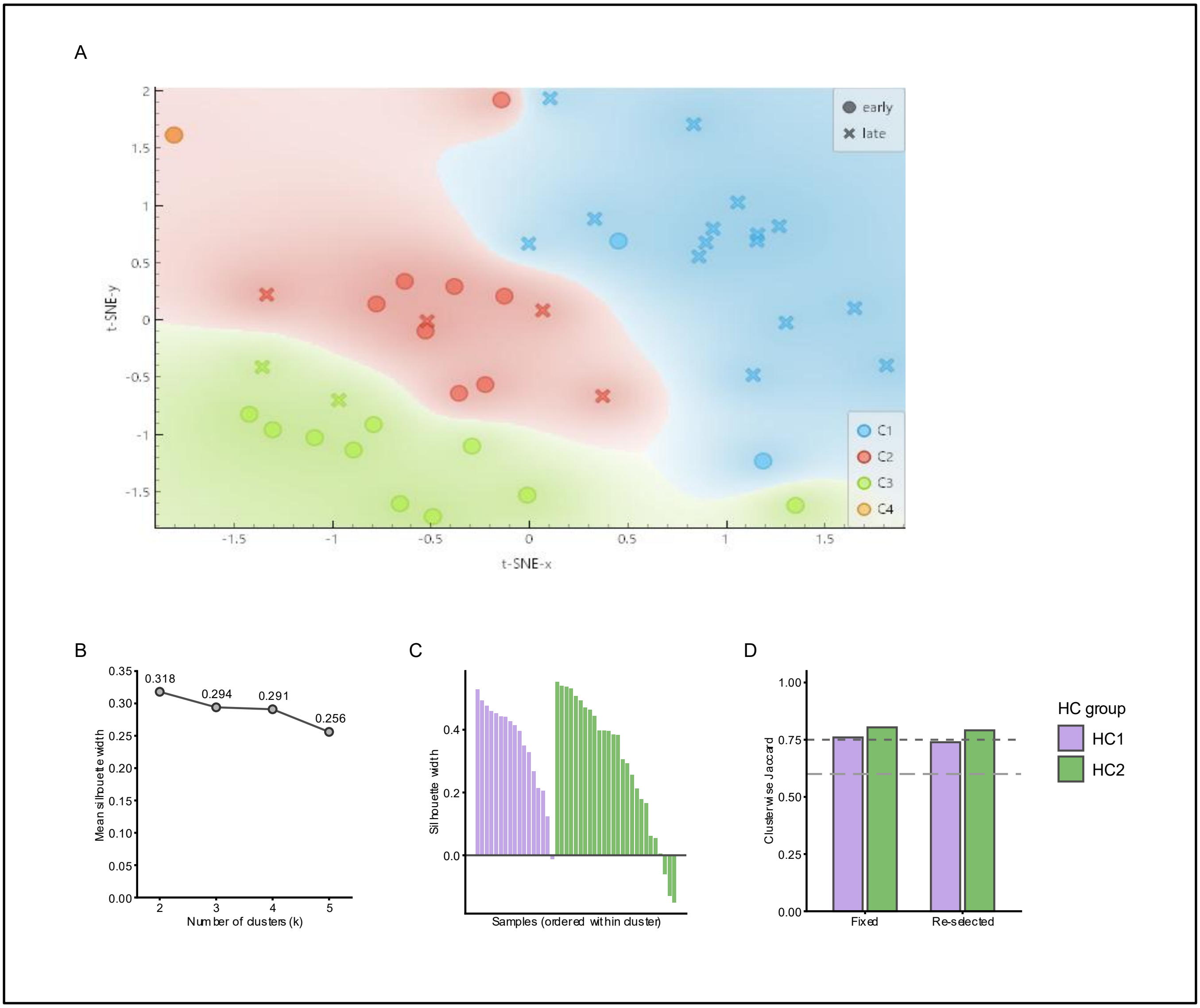

A
early
late
C1
C2
C3
C4
t-SNE-y
t-SNE-x
B
Mean silhouette width
Number of clusters (k)
0.318
0.294
0.291
0.256
C
Silhouette width
Samples (ordered within cluster)
D
Clusterwise Jaccard
Fixed
Re-selected
HC group
HC1
HC2
