# Supplementary material for: Stage‐Associated Collagen Gene Expression and Macrophage‐Associated Immune Features in Nail Unit Melanoma
Source: Pigment Cell Melanoma Res. 2026 Nov 11;39(6):e70143. doi: 10.1111/pcmr.70143 (PMC13755139; doi:10.1111/pcmr.70143)
Supplement: Supplementary file 4 [file PCMR-39-0-s004.pdf]

Figure S10

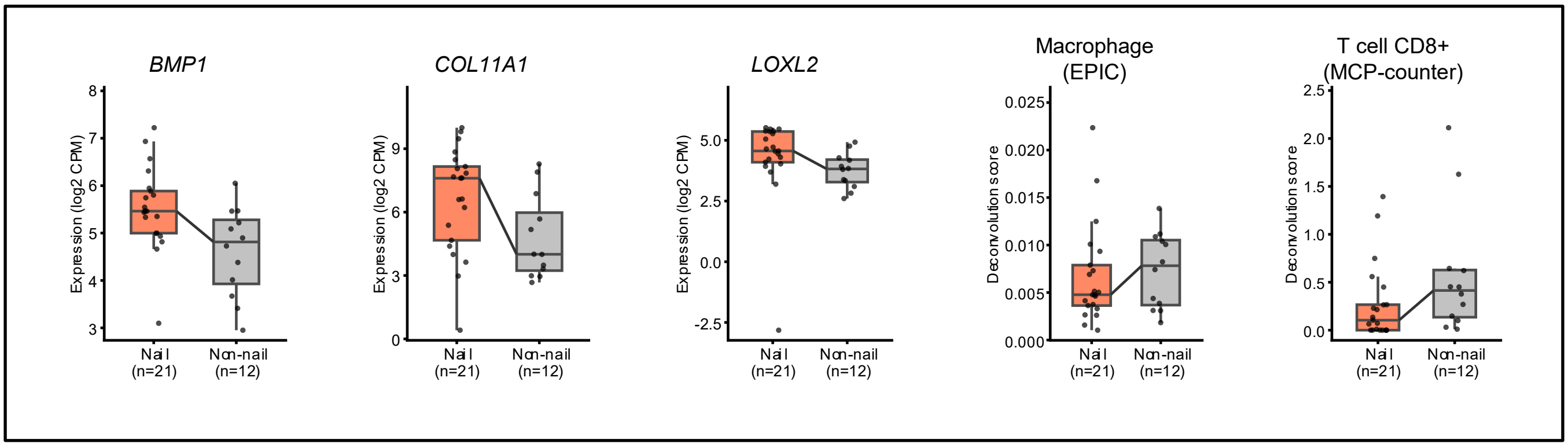

BMP1
COL11A1
LOXL2
Macrophage (EPIC)
T cell CD8+ (MCP-counter)
Expression (log2 CPM)
Deconvolution score
Nail (n=21)
Non-nail (n=12)
