# Supplementary material for: Stage‐Associated Collagen Gene Expression and Macrophage‐Associated Immune Features in Nail Unit Melanoma
Source: Pigment Cell Melanoma Res. 2026 Nov 11;39(6):e70143. doi: 10.1111/pcmr.70143 (PMC13755139; doi:10.1111/pcmr.70143)
Supplement: Supplementary file 5 [file PCMR-39-0-s006.pdf]

# Figure S4

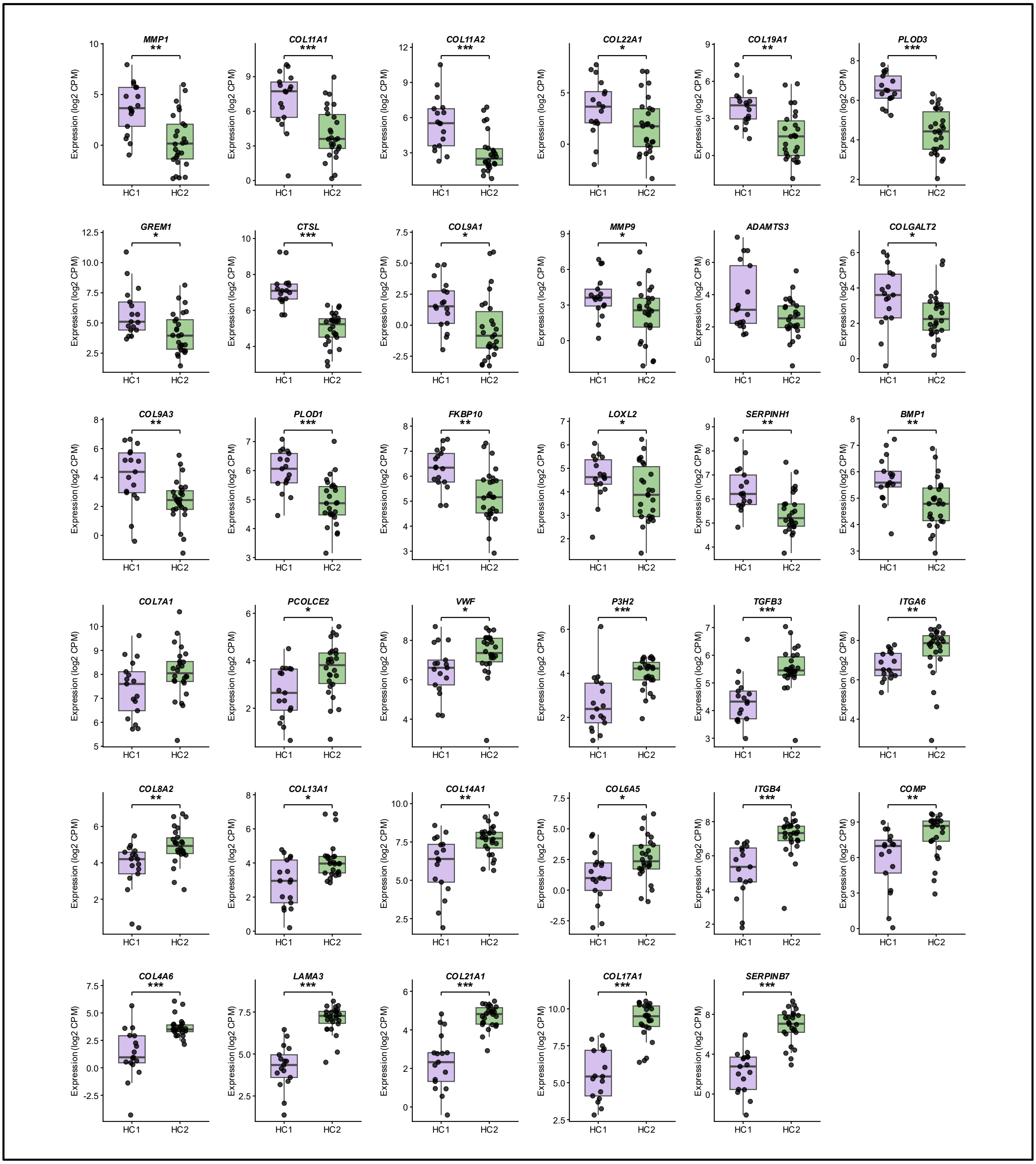

MMP1 **
COL11A1 ***
COL11A2 ***
COL22A1 *
COL19A1 **
PLOD3 ***
GREM1 *
CTSL ***
COL9A1 *
MMP9 *
ADAMTS3
COLGALT2 *
COL9A3 **
PLOD1 ***
FKBP10 **
LOXL2 *
SERPINH1 **
BMP1 **
COL7A1
PCOLCE2 *
VWF *
P3H2 ***
TGFB3 ***
ITGA6 **
COL8A2 **
COL13A1 *
COL14A1 **
COL6A5 *
ITGB4 ***
COMP **
COL4A6 ***
LAMA3 ***
COL21A1 ***
COL17A1 ***
SERPINB7 ***
Expression (log2 CPM)
HC1
HC2
