# Supplementary material for: Stage‐Associated Collagen Gene Expression and Macrophage‐Associated Immune Features in Nail Unit Melanoma
Source: Pigment Cell Melanoma Res. 2026 Nov 11;39(6):e70143. doi: 10.1111/pcmr.70143 (PMC13755139; doi:10.1111/pcmr.70143)
Supplement: Supplementary file 6 [file PCMR-39-0-s009.pdf]

Figure S5

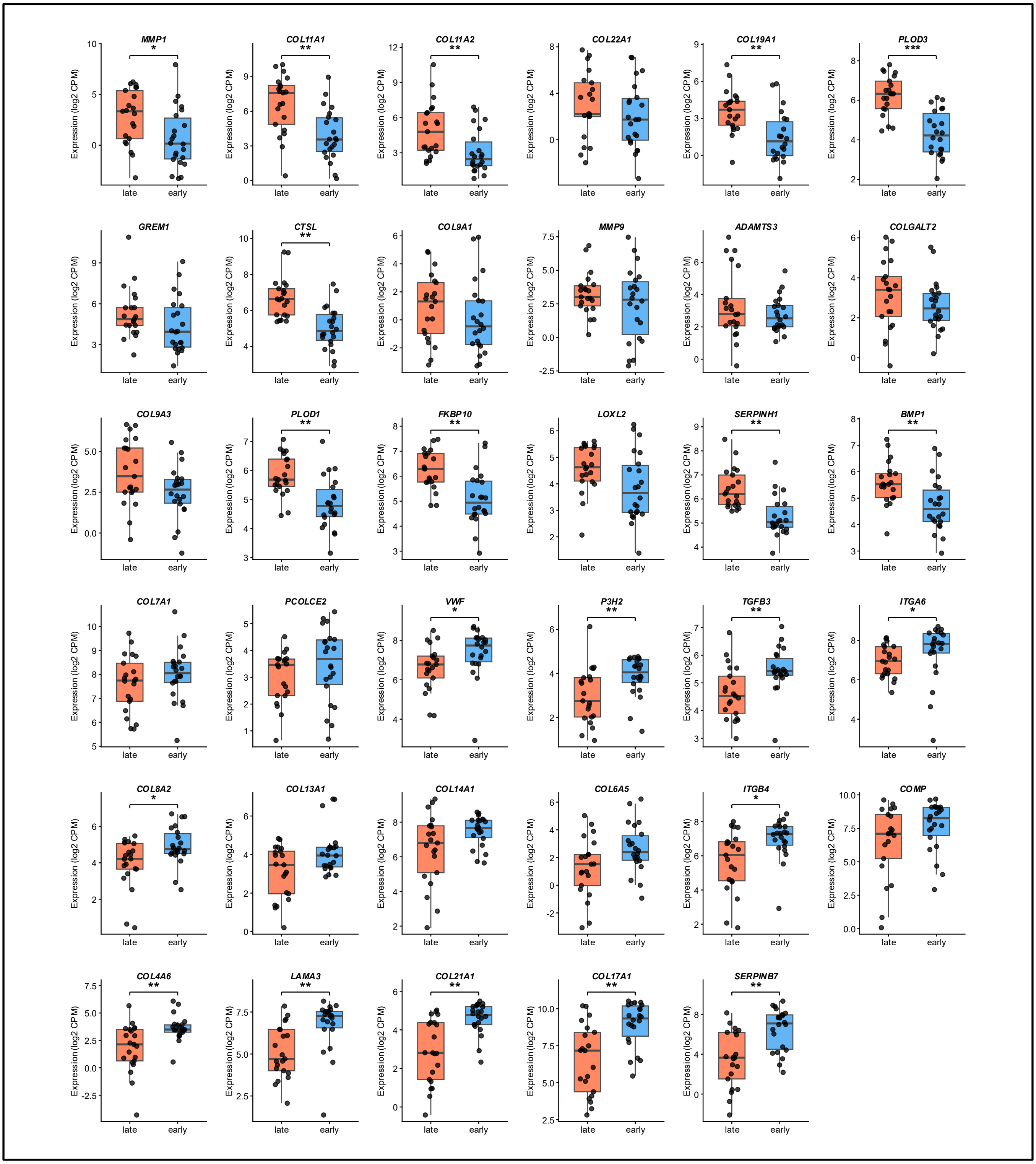
MMP1 *
COL11A1 **
COL11A2 **
COL22A1
COL19A1 **
PLOD3 ***
GREM1
CTSL **
COL9A1
MMP9
ADAMTS3
COLGALT2
COL9A3
PLOD1 **
FKBP10 **
LOXL2
SERPINH1 **
BMP1 **
COL7A1
PCOLCE2
VWF *
P3H2 **
TGFB3 **
ITGA6 *
COL8A2 *
COL13A1
COL14A1
COL6A5
ITGB4 *
COMP
COL4A6 **
LAMA3 **
COL21A1 **
COL17A1 **
SERPINB7 **
Expression (log2 CPM)
late
early
