# Supplementary material for: Stage‐Associated Collagen Gene Expression and Macrophage‐Associated Immune Features in Nail Unit Melanoma
Source: Pigment Cell Melanoma Res. 2026 Nov 11;39(6):e70143. doi: 10.1111/pcmr.70143 (PMC13755139; doi:10.1111/pcmr.70143)
Supplement: Supplementary file 7 [file PCMR-39-0-s014.pdf]

Figure S2

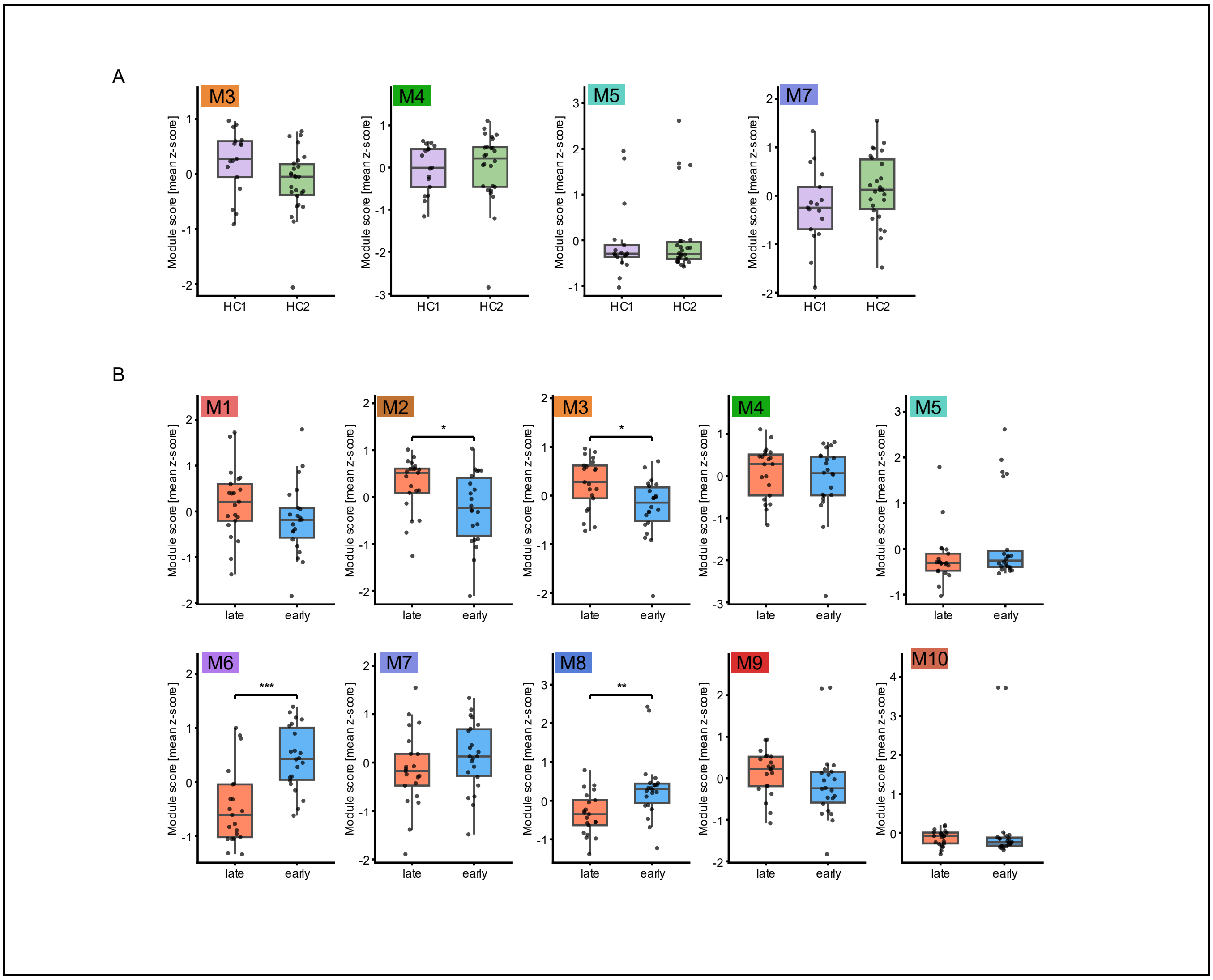

A
M3
M4
M5
M7
Module score [mean z-score]
HC1
HC2
B
M1
M2
M3
M4
M5
M6
M7
M8
M9
M10
late
early
*
**
***
