# Supplementary material for: Stage‐Associated Collagen Gene Expression and Macrophage‐Associated Immune Features in Nail Unit Melanoma
Source: Pigment Cell Melanoma Res. 2026 Nov 11;39(6):e70143. doi: 10.1111/pcmr.70143 (PMC13755139; doi:10.1111/pcmr.70143)
Supplement: Supplementary file 8 [file PCMR-39-0-s016.pdf]

Figure S8

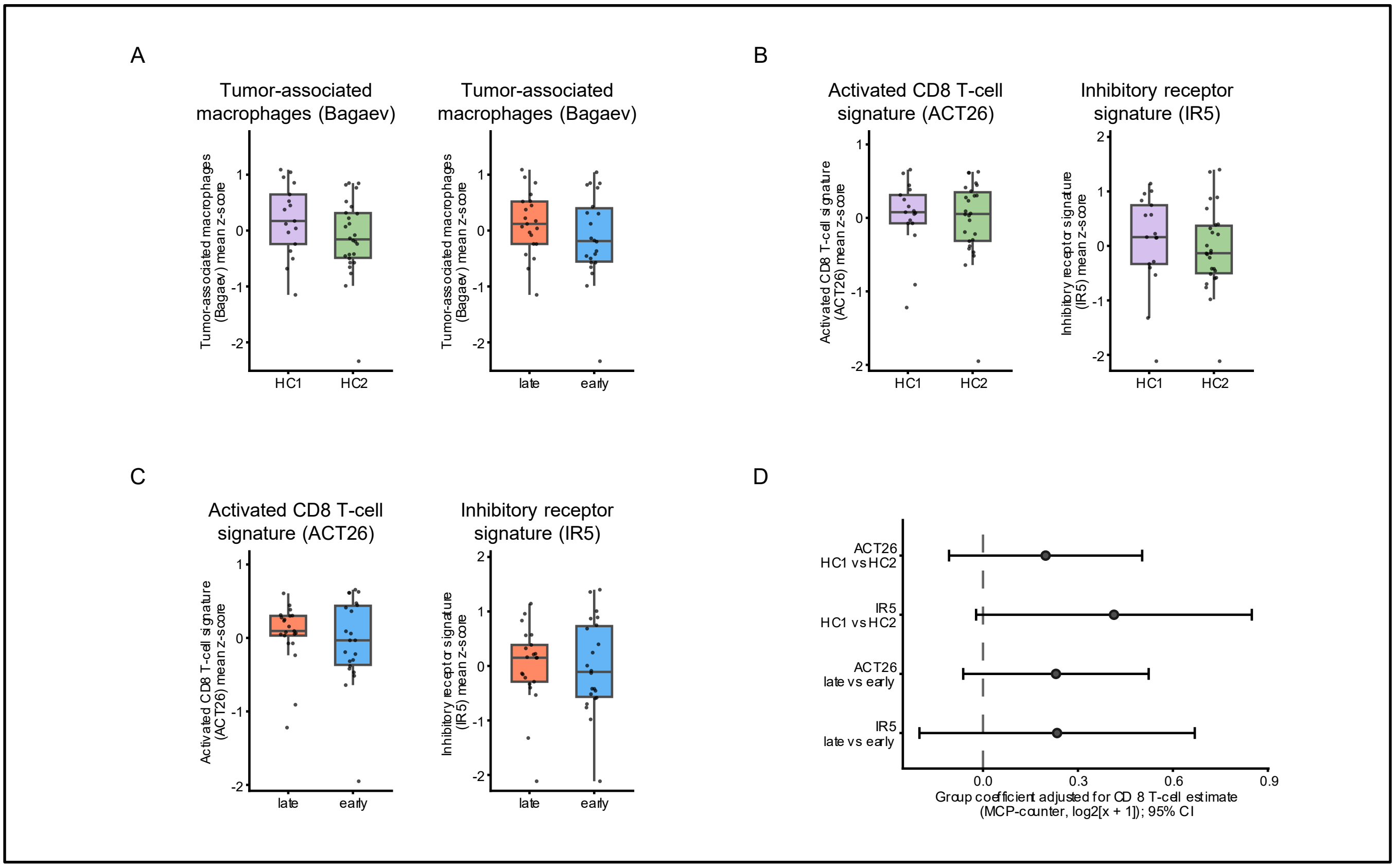

A
Tumor-associated macrophages (Bagaev)
Tumor-associated macrophages (Bagaev) mean z-score
HC1
HC2
Tumor-associated macrophages (Bagaev)
late
early
B
Activated CD8 T-cell signature (ACT26)
Activated CD8 T-cell signature (ACT26) mean z-score
HC1
HC2
Inhibitory receptor signature (IR5)
Inhibitory receptor signature (IR5) mean z-score
C
Activated CD8 T-cell signature (ACT26)
late
early
Inhibitory receptor signature (IR5)
D
ACT26 HC1 vs HC2
IR5 HC1 vs HC2
ACT26 late vs early
IR5 late vs early
0.0
0.3
0.6
0.9
Group coefficient adjusted for CD 8 T-cell estimate (MCP-counter, log2[x + 1]); 95% CI
