# Supplementary material for: Stage‐Associated Collagen Gene Expression and Macrophage‐Associated Immune Features in Nail Unit Melanoma
Source: Pigment Cell Melanoma Res. 2026 Nov 11;39(6):e70143. doi: 10.1111/pcmr.70143 (PMC13755139; doi:10.1111/pcmr.70143)
Supplement: Supplementary file 9 [file PCMR-39-0-s017.pdf]

Figure S3

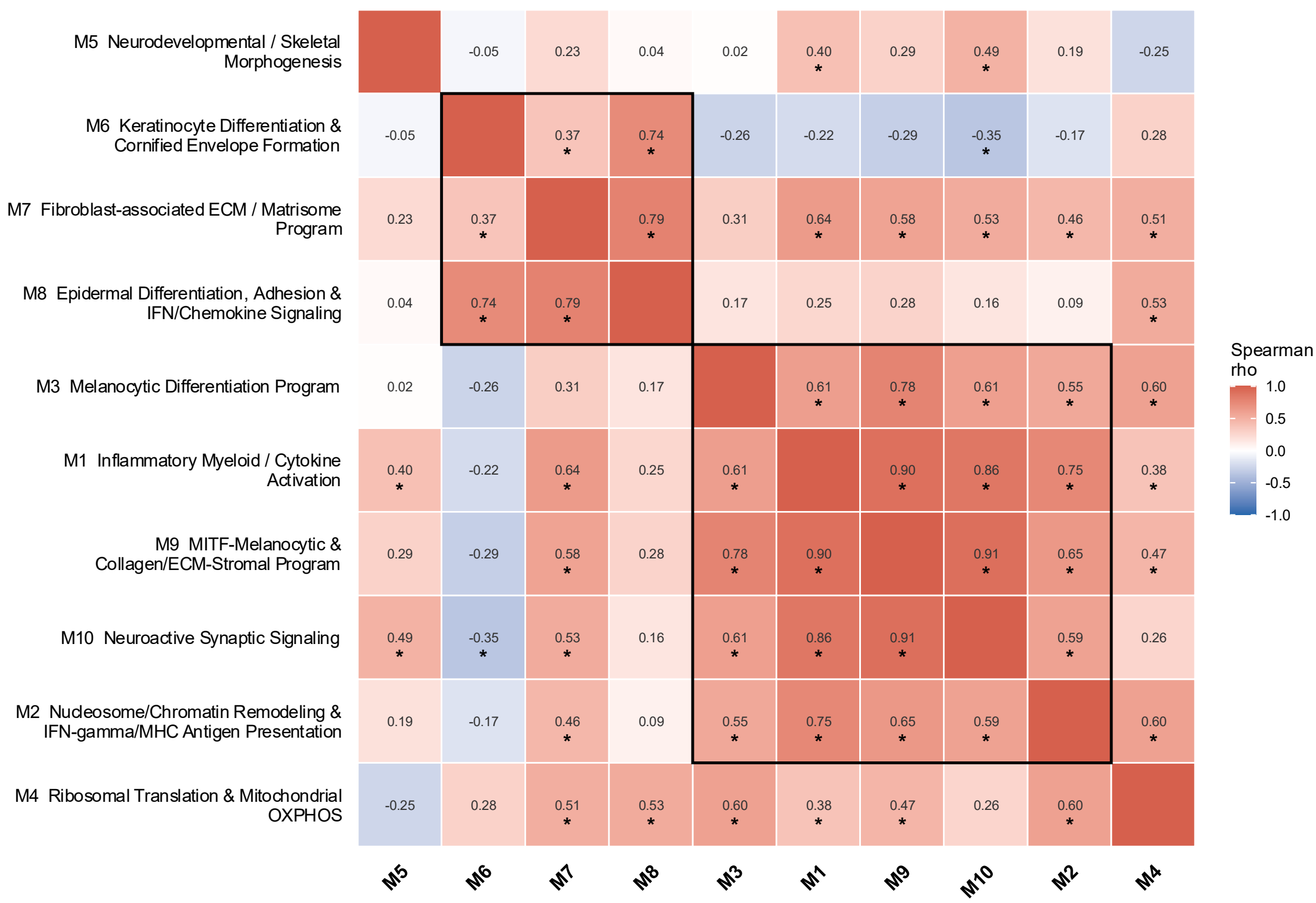
M5 Neurodevelopmental / Skeletal Morphogenesis
M6 Keratinocyte Differentiation & Cornified Envelope Formation
M7 Fibroblast-associated ECM / Matrisome Program
M8 Epidermal Differentiation, Adhesion & IFN/Chemokine Signaling
M3 Melanocytic Differentiation Program
M1 Inflammatory Myeloid / Cytokine Activation
M9 MITF-Melanocytic & Collagen/ECM-Stromal Program
M10 Neuroactive Synaptic Signaling
M2 Nucleosome/Chromatin Remodeling & IFN-gamma/MHC Antigen Presentation
M4 Ribosomal Translation & Mitochondrial OXPHOS
M5 M6 M7 M8 M3 M1 M9 M10 M2 M4
Spearman rho
1.0
0.5
0.0
-0.5
-1.0
