# Supplementary material for: Stage‐Associated Collagen Gene Expression and Macrophage‐Associated Immune Features in Nail Unit Melanoma
Source: Pigment Cell Melanoma Res. 2026 Nov 11;39(6):e70143. doi: 10.1111/pcmr.70143 (PMC13755139; doi:10.1111/pcmr.70143)
Supplement: Supplementary file 10 [file PCMR-39-0-s019.pdf]

Figure S6

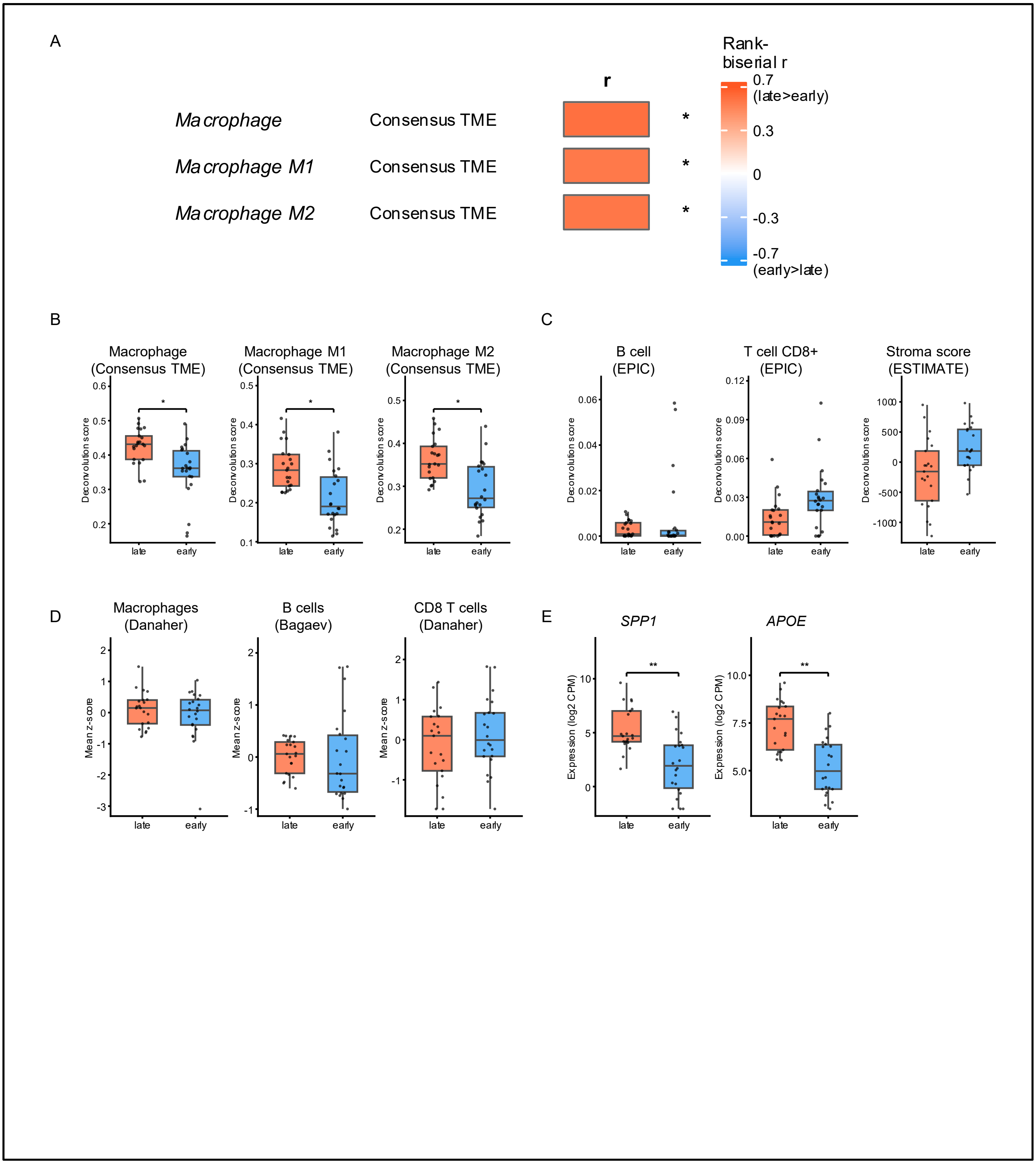
A
r
Rank-biserial r
0.7 (late>early)
0.3
0
-0.3
-0.7 (early>late)
Macrophage
Consensus TME
*
Macrophage M1
Consensus TME
*
Macrophage M2
Consensus TME
*
B
Macrophage (Consensus TME)
Macrophage M1 (Consensus TME)
Macrophage M2 (Consensus TME)
Deconvolution score
late
early
C
B cell (EPIC)
T cell CD8+ (EPIC)
Stroma score (ESTIMATE)
D
Macrophages (Danaher)
B cells (Bagaev)
CD8 T cells (Danaher)
Mean z-score
E
SPP1
APOE
Expression (log2 CPM)
**
